# Metabolic reprogramming driven by EZH2 inhibition depends on cell–matrix interactions

**DOI:** 10.1016/j.jbc.2023.105485

**Published:** 2023-11-20

**Authors:** Teresa W-M Fan, Jahid M.M. Islam, Richard M. Higashi, Penghui Lin, Christine F. Brainson, Andrew N. Lane

**Affiliations:** 1 Center for Environmental and System Biochemistry, University of Kentucky, Lexington, Kentucky, USA; 2 Department of Toxicology and Cancer Biology, University of Kentucky, Lexington, Kentucky, USA; 3 Markey Cancer Center, University of Kentucky, Lexington, Kentucky, USA

**Keywords:** Stable isotope-resolved metabolomics, glucose/glutamine metabolism, extracellular matrix, spheroids, EZH2

## Abstract

EZH2 (Enhancer of Zeste Homolog 2), a subunit of Polycomb Repressive Complex 2 (PRC2), catalyzes the trimethylation of histone H3 at lysine 27 (H3K27me3), which represses expression of genes. It also has PRC2-independent functions, including transcriptional coactivation of oncogenes, and is frequently overexpressed in lung cancers. Clinically, EZH2 inhibition can be achieved with the FDA-approved drug EPZ-6438 (tazemetostat). To realize the full potential of EZH2 blockade, it is critical to understand how cell-cell/cell-matrix interactions present in 3D tissue and cell culture systems influences this blockade in terms of growth-related metabolic functions. Here, we show that EZH2 suppression reduced growth of human lung adenocarcinoma A549 cells in 2D cultures but stimulated growth in 3D cultures. To understand the metabolic underpinnings, we employed [^13^C_6_]-glucose stable isotope-resolved metabolomics to determine the effect of EZH2 suppression on metabolic networks in 2D *versus* 3D A549 cultures. The Krebs cycle, neoribogenesis, γ-aminobutyrate metabolism, and salvage synthesis of purine nucleotides were activated by EZH2 suppression in 3D spheroids but not in 2D cells, consistent with the growth effect. Using simultaneous ^2^H_7_-glucose + ^13^C_5_,^15^N_2_-Gln tracers and EPZ-6438 inhibition of H3 trimethylation, we delineated the effects on the Krebs cycle, γ-aminobutyrate metabolism, gluconeogenesis, and purine salvage to be PRC2-dependent. Furthermore, the growth/metabolic effects differed for mouse Matrigel *versus* self-produced A549 extracellular matrix. Thus, our findings highlight the importance of the presence and nature of extracellular matrix in studying the function of EZH2 and its inhibitors in cancer cells for modeling the *in vivo* outcomes.

EZH2 (Enhancer of Zeste Homolog 2) is a subunit of polycomb repressive complex 2 (PRC2), that catalyzes the trimethylation of histone H3 at lysine 27 (H3K27me3), which represses expression of genes such as *GSK3B*, *TP53* (tumor suppressors) (1) and *CXCR4* (tumor-promoter) (2). EZH2 also has PRC2-independent functions including transcriptional coactivation of oncogenic proteins (*e.g.* cyclin D1, NFκB) and methylation of nonhistone proteins (*e.g.* STAT3 and talin) (3, 4). EZH2 is critical to cancer initiation/progression and is frequently overexpressed in lung cancer and many other human cancers (3, 5, 6, 7). From our in-house RNAseq data, we found that 60 out of 66 patients’ non-small cell lung cancer tissues had overexpressed EZH2 mRNA relative to the matched noncancerous lung tissues (average non-small cell lung cancer/noncancerous ratio of 6.27 ± 1.23).

Recent evidence points to an important role of EZH2 in promoting tumorigenesis and progression by reprogramming cancer cell metabolism. EZH2 was found to activate aerobic glycolysis in glioblastomas (8), esophageal cancer (9), and prostate cancer cells (10) to promote tumorigenesis and progression. EZH2 blockade has also been shown to have a widespread impact on the metabolic profile of melanoma cells, reflecting suppressed Krebs cycle/amino acid metabolism and stimulated lipid synthesis (11). On the other hand, EZH2 inhibition enhanced lipoprotein-mediated lipid accumulation by promoting APOE expression in adipocytes (12). In glioblastoma, silencing EZH2 with siRNA resulted in a decline in fatty acid synthase levels, which was accompanied by lower fatty acid levels (13). Suffice to say, our knowledge of EZH2 modulation of anabolic metabolic networks to influence cancer cell growth is still at its infancy.

Of concern is that prior understanding of EZH2’s function has been primarily derived from 2D cell cultures, which fail to recapitulate the tumor microenvironment (TME), namely lacking cell-cell and cell-matrix interactions, nutrient/O_2_ gradients, and other TME-related stresses (14, 15). All these factors induce metabolic and other functional plasticity in the TME. As a result, most therapeutic agents showing promise in 2D monolayer cultures fail to carry the effect *in vivo* (16). Most recently, Chen *et al*. reported that murine 2D lung cancer cultures had significantly reduced gene expression related to lung lineage determination compared to 3D tumoroids. The latter retained transcriptional programs specific to lung tissues, indicating that 3D cultures preserve more of the molecular features of the original tissue. They also found that *Ezh2* null 2D cells displayed enriched expression of PRC2 target genes, EMT, and immunity-related signatures relative to the WT counterparts, while the opposite was evident in 3D tumor spheroids (17). Moreover, enhanced gene expression of *Foxp2*, a transcription factor that promotes migration and stemness, was shown in *Ezh2* null *versus* WT tumoroids, which was not evident in the 2D cell case. However, it is unknown if and how cell–cell interactions and extracellular matrix (ECM) in the TME influences EZH2’s function in cancer cells. It is also unclear whether blocking EZH2 will have a better cancer treatment outcome *in vivo*, despite the promise of cancer suppression demonstrated in 2D cancer models (18, 19, 20). As a result, although several EZH2 inhibitors (EZH2i) have been developed as therapeutic candidates for cancer and a highly specific EZH2i EPZ-6438 (tazemetostat) was approved by the FDA in 2020 as a standalone treatment for epithelioid sarcoma (21), such use of EZH2i for treating solid tumors has not been widely accepted (11).

To address the question of cell–cell interaction and ECM effect on EZH2’s function, we utilized [^13^C_6_]-glucose as tracer and stable isotope-resolved metabolomic (SIRM) approach to examine the effect of EZH2 suppression on the metabolic networks in human lung adenocarcinoma A549 cells, grown as 2D cultures or 3D spheroids. We then applied dual tracers [^2^H_7_]-glucose plus [^13^C_5_,^15^N_2_]-Gln and SIRM analysis to delineate the metabolic outcomes of inhibiting EZH2’s trimethyltransferase activity with EPZ-6438 in 3D spheroids formed with self-produced ECM or with mouse Matrigel. We found that growth was stimulated in 3D A549 spheroids but inhibited in 2D cells in response to EZH2 knockdown (KD). This was accompanied by activated canonical and anaplerotic Krebs cycle activity, neoribogenesis and salvage synthesis of ATP in 3D but not in 2D cells. We also found that synthesis of γ-aminobutyrate (GAB) was activated in 3D but not in 2D cells, which is likely to impact cancer cell interactions with other cell types *via* GAB signaling in the TME. We further observed Gln-fueled gluconeogenesis (GNG) and preferred diversion of GNG products to the pentose phosphate pathway and nucleotide synthesis in 3D spheroids. Activation of these anabolic processes and growth stimulation by EPZ-6438 depended on the nature of ECM, that is, Matrigel *versus* A549’s own ECM. Thus, cell–cell and cell–matrix interactions had a profound impact on EZH2’s function and downstream metabolic phenotype in A549 cells.

## Results

### EZH2 suppression enhances growth in 3D lung cancer A549 spheroids but attenuates growth in the corresponding 2D cell cultures

We first evaluated the effect of EZH2 KD on human lung adenocarcinoma A549 cell growth, as 2D and 3D spheroid cultures in a 384-well plate. Suppression of EZH2 protein expression in 2D A549 cells was achieved by transduction with viruses encoding two different short-hairpin (sh) sequences targeting *EZH2*, A6 and A9 (referred therein as A6 and A9 cells) or a control *shGFP* virus. *EZH2* KD in A549 cells was verified by western blotting, with the A6 cells showing a greater reduction in EZH2 protein than A9 cells (Fig. S1*A*). However, suppression of the epigenetic writer function of EZH2, i.e. histone H3 trimethylation at lysine 27 (H3K27me3) by A6 and A9 relative to the control (*shGFP*) (referred as *shGFP* cells) was similar (Fig. S1*A*). Cell growth was measured as the fluorescence of PrestoBlue (PB) normalized to that of live cell nuclear stain Hoechst (H). Fig. S1*B* showed that EZH2 KD led to reduced 2D cell growth with both A6 and A9 cultures. In contrast, A6 cells enhanced growth while the A9 cells had no change in 3D A549 spheroid growth in Matrigel. The former agreed with the result of a previous report (6). The PB-based growth effect was confirmed by protein analysis of the corresponding 2D and 3D cell cultures in a 24-well plate (Fig. S1*C*). EZH2 KD also attenuated colony formation (Fig. S1*E*) but stimulated cell migration (Fig. S1*D*) with little effect on cell invasion (Fig. S1*F*) in 2D cultures. In contrast, the 3D spheroids showed reduced migration + invasion in response to EZH2 KD (Fig. S1*G*), which again differed from the response of the 2D counterpart.

Moreover, the two shEZH2 cell lines had different morphologies in Matrigel, with A9 spheroids forming more condensed ring-like structures while A6 spheroids displayed less coalesced structures than the control *shGFP* spheroids (Fig. S1*H*). These data suggest that cell-cell interactions and the presence of ECM have a profound influence on EZH2’s capacity for modulating cancer cell growth, migration, and/or invasion.

### EZH2 suppression differentially reprograms lung cancer cell metabolism in 2D cultures *versus* 3D spheroids

As cell metabolism is required for cell growth, we determined how EZH2 KD modulated A549 cell metabolism in 2D cells *versus* 3D spheroids. We employed [^13^C_6_]-glucose as tracer with stable isotope-resolved metabolomic (SIRM) analysis to broadly track changes in metabolic networks. The networks traced included glycolysis, the Krebs cycle, the pentose phosphate pathway (PPP), and purine nucleotide metabolism.

### EZH2 KD distinctly alters glycolysis, the Krebs cycle, and Krebs cycle-related metabolism in 2D *versus* 3D cultures of A549 cells

**Figure 1:**
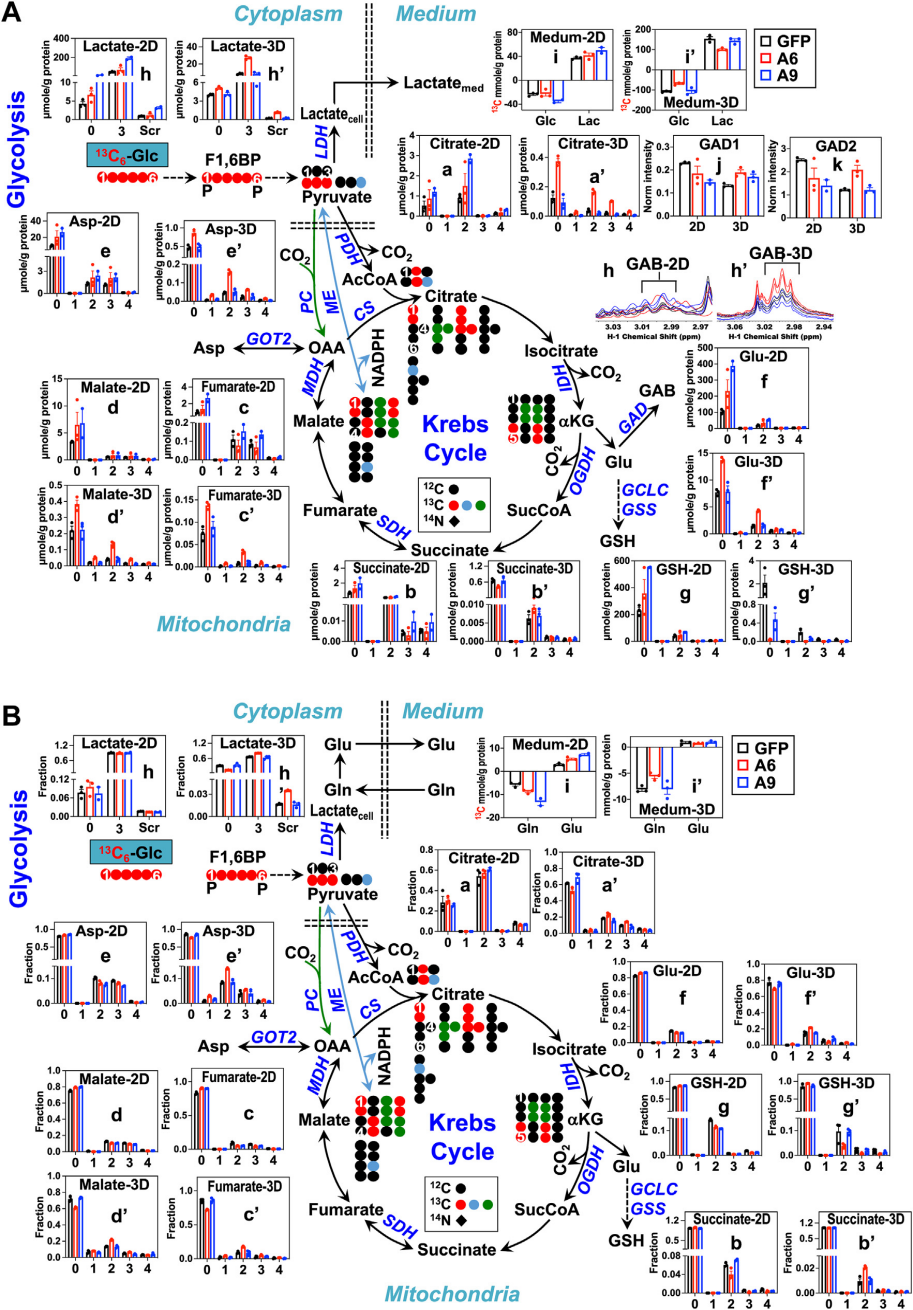
***EZH2* suppression inhibits glycolysis and enhances the Krebs cycle and GAB metabolism in 3D A549 spheroids but not in 2D cells.** A549 cells with shGFP (****), A6 (), or A9 () virus were grown as 2D cultures (a-i) or 3D spheroids (a’-i’) in Lunagel in the presence of ^13^C_6_-Glc (n = 3; each as biological replicate). ^13^C labeling of glycolytic and Krebs cycle metabolites were quantified as μmole/g protein (*A*) or fractional enrichment (*B*) with ^1^H NMR (h-h’, *A*) and IC-UHR-FTMS (the rest), as described in Experimental procedures. The atom-resolved pathway scheme tracks the fate of ^13^C atoms from the Glc tracer in glycolysis and the Krebs cycle. •: ^12^C; , , : ^13^C derived from pyruvate dehydrogenase (PDH), pyruvate carboxylase (PC), and malic enzyme (ME)-initiated Krebs cycle reactions. *Solid*, *dashed*, and double-headed arrows depict single, multiple, and reversible reactions, respectively; *double dashed lines* delineate subcellular compartments; numbers in X-axis denote the number of ^13^C atoms. Scr, scrambled; AcCoA/SucCoA, acetyl/succinyl coenzyme A; OAA, oxaloacetate; GSH, glutathione; LDH/IDH/OGDHSDH/MDH: lactate/isocitrate/oxoglutarate/succinate/malate dehydrogenase; CS, citrate synthase; GAD, glutamate decarboxylase; GOT2, glutamic-oxaloacetic transaminase 2; GCLC, glutamate-cysteine ligase catalytic subunit; GSS, glutathione synthetase. See Tables S2 and S3 for the statistics of changes in metabolites and their isotopologs. EZH2, Enhancer of Zeste Homolog 2; GAB, γ-aminobutyrate.

We tracked the transformation of [^13^C_6_]-glucose (^13^C_6_-Glc) into the glycolytic fermentation product ^13^C-lactate and its subsequent release into the medium (^13^C-Lactate_med_) (Fig. 1*A*). As the level of ^13^C-Lactate_med_ dominated over that of the intracellular ^13^C-lactate (^13^C-Lactate_cell_), it is a more robust indicator of overall glycolytic activity. We observed an insignificant effect in the A6 cells () but enhancement in the A9 cells () on the buildup of both ^13^C-Lactate_med_ (k) and Lactate_cell_ in the 2D cultures (j), which is comparable to the effect on the consumption of the parent glucose tracer. Together, these data indicate that A6 cells had no changes in glycolysis but A9 cells had activated glycolysis in 2D culture. In contrast, A6 spheroids had attenuated uptake of ^13^C-Glc and the buildup of ^13^C-Lactate_med_ in 3D spheroids (k’), whereas A9 spheroids had no change in glycolysis. Further, the amount of ^13^C-Lactate excreted and ^13^C-Glc consumed was much higher in 3D than 2D cells, which suggests enhanced glycolytic capacity on forming the spheroids. As for the ^13^C_3_- (3) and ^13^C scrambled (scr) isotopologues of Lactate_cell_, the level (j, Fig. 1*A*) increased without changes in the fractional enrichment (h, Fig. 1*B*) in 2D A9 cells while both levels and fractional enrichment of the corresponding isotopologues showed insignificant changes in 2D A6 cells, compared with 2D *shGFP* cells (). Such ^13^C scrambling in lactate can result from ^13^C_6_-Glc metabolism *via* the PPP and/or gluconeogenesis. These changes in ^13^C labeling patterns of Lactate_cell_ agree with those of Lactate_med_. In contrast, both ^13^C_3_- and ^13^C-scrambled (scr)-Lactate_cell_ increased in both level and fractional enrichment in the A6 spheroids but showed no changes in A9 spheroids, compared with 3D *shGFP* spheroids (j’, Fig. 1*A*; h’, Fig. 1*B*). The changes in ^13^C labeling patterns of Lactate_cell_ were opposite to those of Lactate_med_ in A6 spheroids (i’, Fig. 1*A*), which points to a block in lactate export and/or cellular turnover. This result also illustrates the unreliability of Lactate_cell_ as a marker for glycolytic activity, and Lactate_med_ must be analyzed to determine the effect on glycolysis. Moreover, the glycolytic reprogramming induced by EZH2 KD could not account for the changes in cell growth (Fig. S1, *B* and *C*).

Other than conversion to ^13^C_3_-lactate, the glycolytic end product ^13^C_3_-pyruvate can be oxidized to ^13^C_2_-acetyl CoA before entering the Krebs cycle to respectively produce ^13^C_2_- and ^13^C_4_-citrate in the first and second turns of the cycle *via* the pyruvate dehydrogenase (PDH) (•)-initiated reactions (Fig. 1). ^13^C_3_-pyruvate may also be converted to ^13^C_3_-citrate/Asp *via* the pyruvate carboxylase (PC)-initiated reactions (•), which replenishes oxaloacetate and thus Krebs cycle intermediates that are diverted for anabolic uses such as fueling lipid (*via* citrate) and nucleotide (*via* Asp) synthesis. Moreover, ^13^C_3_-pyruvate can be metabolized *via* the Krebs cycle to ^13^C_2_-3,4-malate, which is converted to ^13^C_1_-3-malate (•) *via* the reversible malic enzyme (ME) reaction. This reaction can also replenish the Krebs cycle while producing NADPH. We saw increased buildup of ^13^C_2_-citrate (a), ^13^C_2_/^13^C_3_-Asp (e), and to a lesser extent ^13^C_2_-fumarate (c), and -malate (d) (Fig. 1*A*), with no change (citrate) or decrease (Asp, malate, fumarate) in the corresponding fractional enrichment (Fig. 1*B*) in 2D A9 *versus shGFP* cells. These patterns of changes point to increased unlabeled pool(s) of citrate, Asp, malate, and fumarate despite their enhanced synthesis *via* PDH and PC reactions. The unlabeled pool(s) derived from the metabolism of non-labeled precursor(s) such as oxidation of Gln. The changes in ^13^C labeling of these metabolites were less significant in 2D A6 cells. In contrast, compared with 3D *shGFP* spheroids, A6 spheroids showed enhanced levels and fractional enrichments of the ^13^C_2_- and ^13^C_3_-isotopologues of these metabolites while A9 spheroids had no changes in these labeling patterns (a’-e’, Fig. 1, *A* and *B*). Likewise, a similar change was evident for ^13^C_1_-malate, -fumarate, -citrate, and -Asp. These results suggest enhanced Krebs cycle activity *via* PDH/PC/ME-initiated reactions but attenuated oxidation of the unlabeled source(s) in A6 but not in A9 spheroids. The above Krebs cycle reprogramming could account for the increased growth occurring in A6 cells cultured as 3D spheroids, but not growth attenuation in 2D cultures (Fig. S1, *B* and *C*).

We further noted striking differences in the Krebs cycle–related metabolism between 2D cells and 3D spheroids. The steady-state pool of unlabeled (0) and ^13^C labeled (2–4) Glu (f, f’) or its anti-oxidant product glutathione (GSH, g, g’) was respectively >10- and 100-fold higher in 2D cells than 3D spheroids. This could lead to a lower anti-oxidation capacity in 3D than 2D cells. In contrast, another Glu product γ-aminobutyrate (GAB) produced *via* the glutamate decarboxylase reaction was hardly detected by ^1^H NMR in 2D cells (i) but readily observed in 3D spheroids (i’) (Fig. 1*A*). Changes in these metabolites in response to EZH2 KD also differed between 2D cells and 3D spheroids (Fig. 1, *A* and *B*). The Glu response was akin to that of citrate but GSH showed the opposite response, that is, reduced instead of enhanced buildup and fractional enrichment of ^13^C labeled GSH in A6 *versus shGFP* spheroids. Blockage of GSH synthesis from glucose could account for this response of A6 spheroids. Unlabeled GAB’s response (i’) tracked that of unlabeled Glu (f’) while little ^13^C labeled GAB was detected, which indicates that GAB was produced primarily from non-glucose source(s) such as Gln or pre-existing glycogen. The enhanced buildup of GAB by EZH2 suppression is consistent with overexpression of GAD1 (l) and GAD2 (m) in A6 *versus shGFP* spheroids (Fig. 1*A*). The neurotransmitter GAB is known to modulate the TME including anti-inflammatory macrophage differentiation (22). We also recently demonstrated that GAB metabolism and signaling promoted differentiation of T_H_17 and regulatory T cells (23). Moreover, exogenous GAB has been shown to inhibit the growth of 2D lung cancer cells including A549 cells (24). Induction of GAB production in 3D A549 spheroids is consistent with a role of GAB role in modulating the TME.

### EZH2 KD activates the pentose phosphate pathway in 2D A9 cells or 3D A6 spheroids

**Figure 2:**
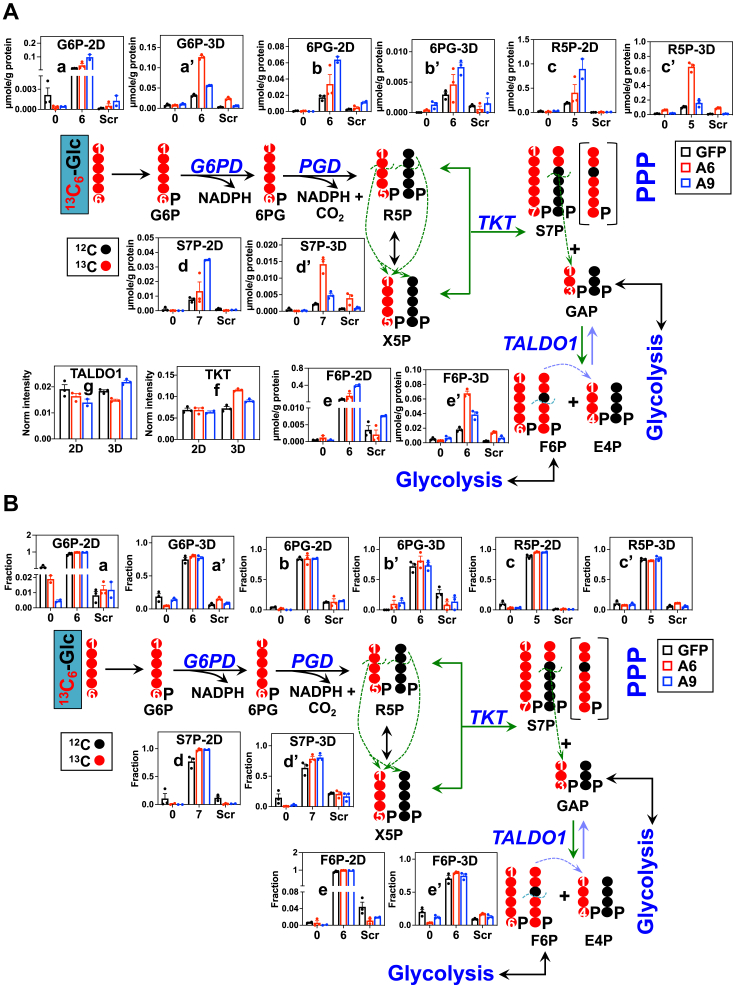
***EZH2* suppression by A9 or A6 vectors activates the pentose phosphate pathway respectively in 2D cultures or 3D spheroids of A549 cells.** The same polar extracts as in Figure 1 were analyzed by IC-UHR-FTMS to obtain μmole/g protein (*A*) or fractional enrichment (*B*) of PPP metabolites (n = 3; each as biological replicate). Single and double-headed *black arrows* denote irreversible and reversible reactions, respectively; solid *green* and light *purple* arrows track the forward and reverse reactions of the nonoxidative branch of PPP; *dashed* arrow and curves depict the transketolase (TKT) and transaldolase (TALDO1) reactions; numbers in X-axis denote the number of ^13^C atoms. Scr, scrambled; G6PD/PGD, glucose-6-phosphate/6-phosphogluconate dehydrogenase; R5P, ribose-5-phosphate; S7P, sedoheptulose-7-phosphate; GAP, glyceraldehyde-3-phosphate; E4P, erythrose-4-phosphate; F6P, fructose-6-phosphate. •: ^12^C; : ^13^C. See Tables S2 and S3 for the statistics of changes in metabolites and their isotopologues. EZH2, Enhancer of Zeste Homolog 2; PPP, pentose phosphate pathway.

In addition to glycolysis, glucose is the key carbon source for the PPP and glycogen synthesis (Fig. 2). PPP produces ribose-5-phosphate (R5P) for fueling nucleotide synthesis and NADPH as reductant for supporting anabolic metabolism and anti-oxidation. Except for 6-phosphogluconate (6PG) in A6 spheroids, we saw similar change patterns of unlabeled and uniformly ^13^C labeled PPP metabolites as those of unlabeled and ^13^C-citrate (Fig.ure 1 A) in 2D cells (a-e) and 3D spheroids (a’-e’) in response to A6 or A9-induced *EZH2* KD (Fig. 2*A*). Namely, enhanced buildup of their levels was evident in 2D A9 cells or in 3D A6 spheroids. However, the fractional enrichment of ^13^C labeled metabolites was not significantly altered by either sh vectors (Fig. 2*B*). The much lower buildup of ^13^C_6_-6PG and high buildup of ^13^C_5_-R5P and ^13^C_7_-S7P in A6 spheroids could result from a block in the phosphogluconate dehydrogenase (PGD) reaction and/or enhanced transketolase (TKT) activity. The latter is consistent with overexpression of TKT protein in A6 *versus shGFP* spheroids (f, Fig. 2*A*). On the other hand, changes in TKT expression could not account for the enhanced buildup of ^13^C_5_-R5P and ^13^C_7_-S7P in 2D A9 cells but suppressed transaldolase (TALDO1, g, Fig. 2*A*) is consistent with such labeling patterns. Overall, these data are consistent with increased glucose metabolism through the PPP or accelerated PPP activity in A9 cells grown in 2D, or in A6 cells grown as 3D spheroids. The latter agrees with the growth stimulation observed for A6 *versus shGFP* spheroids but was in contrast to the growth inhibition seen for 2D A9 *versus shGFP* cells.

In addition to uniformly ^13^C-labeled PPP metabolites, we observed ^13^C-scrambled (Scr) species at higher levels in 3D spheroids than 2D cells. They responded to A6-or A9-induced EZH2 KD similarly as the fully ^13^C labeled species (Fig. 2*A*). These scrambled species are expected to result from the TKT and TALDO1 reactions in the non-oxidative branch of PPP although GNG could also contribute to their formation. Thus, 3D spheroids display a higher capacity for PPP and/or GNG than the 2D counterpart.

### EZH2 KD activates purine nucleotide synthesis in 2D A9 cells or 3D A6 spheroids

**Figure 3:**
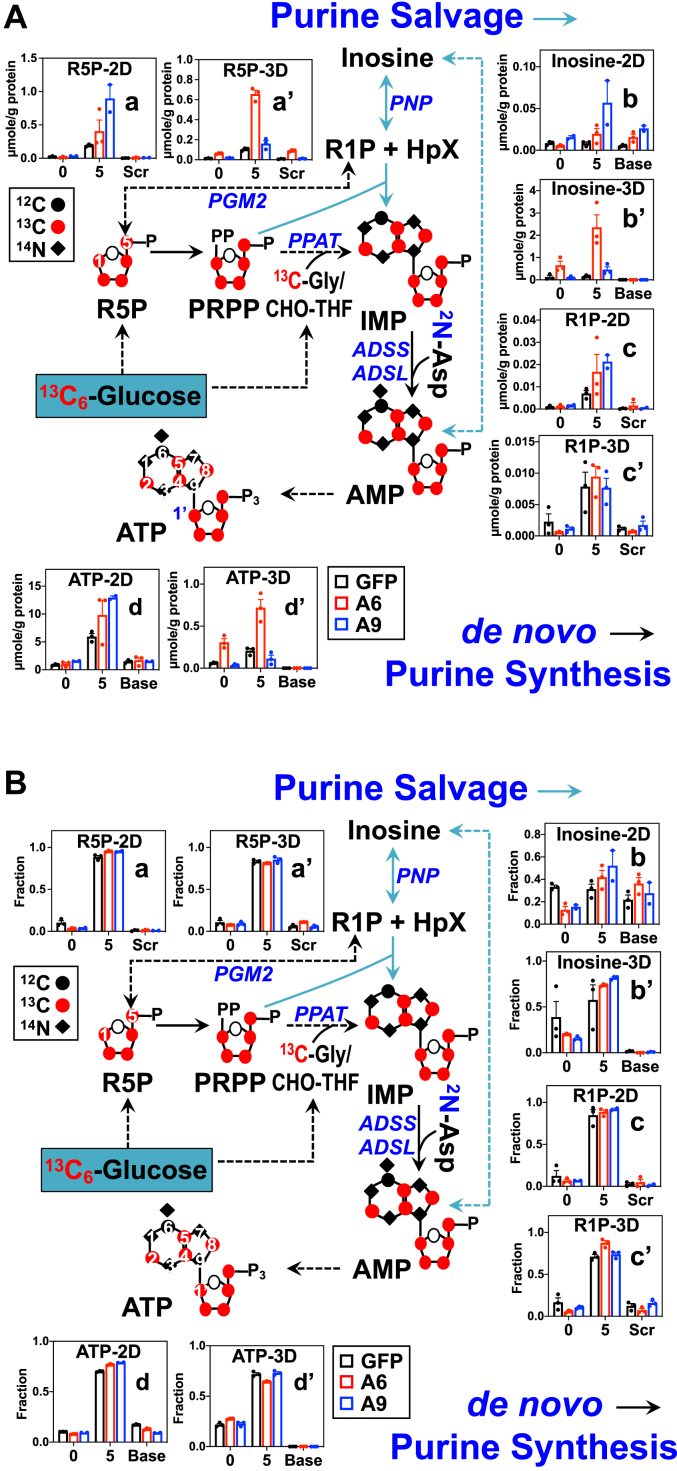
***EZH2* knockdown by A9 or A6 virus activates salvage synthesis of ATP respectively in 2D cultures or 3D spheroids of A549 cells.** The same polar extracts as in Figure 1 were analyzed by IC-UHR-FTMS to obtain μmole/g protein (*A*) or fractional enrichment (*B*) of metabolites in the synthesis pathways of adenine nucleotides (n = 3; each as biological replicate). Single/double-headed solid/*dashed* arrows denote irreversible/reversible single/multiple reactions, respectively; *light blue* arrows track the purine salvage pathway; numbers in X-axis denote the number of ^13^C atoms. Scr, scrambled; R1P, ribose-1-phosphate; HpX, hypoxanthine; PRPP, phosphoribosyl pyrophosphate; CHO-THF, N10-formyltetrahydrofolate; PNP, purine nucleoside phosphorylase; PGM2, phosphoglucomutase 2; PPAT, phosphoribosyl pyrophosphate amidotransferase; ADSS, adenylosuccinate synthase; ADSL, adenylosuccinate lyase. •: ^12^C; ◆: ^14^N; : ^13^C. See Tables S2 and S3 for the statistics of changes in metabolites and their isotopologues. EZH2, Enhancer of Zeste Homolog 2.

Enhanced PPP activity can support increased purine and pyrimidine nucleotide synthesis. We thus tracked the metabolism of ^13^C_6_-Glc through the purine synthesis (*de novo* and salvage) pathways in 2D and 3D A549 cells. Indeed, ^13^C_5_-ribose incorporation into ATP (5, d-d’) displayed the same change trend as that of the precursor ^13^C_5_-R5P (5, a-a’) in both 2D cells and 3D spheroids (Fig. 3, *A* and *B*). This is consistent with PPP activation leading to enhanced synthesis of purine nucleotides in 2D A9 cells and 3D A6 spheroids, relative to their *shGFP* counterparts. We also saw a similar change pattern of ^13^C_5_-inosine (5, b-b’) in 2D A9 and 3D A6 spheroids *versus* respective *shGFP* cells. This suggests an increase in ATP catabolism, as inosine is a catabolite of ATP. However, inosine is also a precursor to the salvage pathway of purine nucleotide synthesis by conversion to hypoxanthine (HpX) and ribose-1-phosphate (R1P). HpX can then react with phosphoribosyl pyrophosphate (PRPP) to form inosine monophosphate (IMP) and ultimately ATP (25). As the salvage pathway utilizes pre-existing adenine, this pathway is expected to produce ATP with much lower carbon incorporation from glucose in the adenine base than in the ribose unit (readily synthesized from PPP). Indeed, we observed lower ^13^C labeling in adenine than that in the ribose subunit in 2D cells and even more so in 3D spheroids (d-d’) (Fig. 3, *A* and *B*). This suggests that ATP in 2D cells was largely produced *via* the salvage pathway, which is even more pronounced in 3D spheroids. In addition, we saw much less enhanced buildup (c’, Fig. 3*A*) but higher fractional enrichment of ^13^C_5_-R1P (c’, Fig. 3*B*) than ^13^C_5_-inosine in A6 *versus shGFP* spheroids. This points to increased conversion of R1P to R5P, which should better support the salvage pathway. Enhanced salvage synthesis of purine nucleotides in A6 spheroids is consistent with its enhanced growth relative to *shGFP* spheroids; however that in 2D A9 cells could not account for its reduced growth relative to 2D *shGFP* cells.

Altogether, cell-cell and/or cell-ECM interactions had a prominent impact on EZH2 modulation of cell metabolism, as also evident for cell growth. Enhanced Krebs cycle/PPP activity and salvage synthesis of purine nucleotides could at least in part support the growth stimulation induced by EZH2 KD in 3D A549 spheroids, but were opposed to growth inhibition in 2D A549 cells.

### Metabolic reprogramming induced by inhibition of the PRC2-dependent pathway in A549 spheroids

As the effect of E*ZH2* KD can be mediated *via* Polycomb Repressive Complex 2 (PRC2), i.e. H3K27me3-dependent and PRC2-independent mechanism (18), we used a specific inhibitor (EPZ-6438 or Tazemetostat) of EZH2 that blocks the formation of H3K27me3 (26) to selectively observe the growth and metabolic outcomes of suppressing the PRC2-dependent pathway in A549 spheroids. As shown in Fig. S1*B*, when grown in Matrigel, A549 spheroids spontaneously coalesced to form larger structures, which varied somewhat from sample to sample. This may have contributed to the relatively large variance in the treatment effect. EPZ-6438 stimulated A549 spheroid growth in Matrigel in a dose-dependent manner and this effect plateaued by 10 μM and higher concentrations (Fig. S2*A*). This growth stimulation is analogous to that observed for the EZH2 KD (A6) spheroids (Fig. S1, *B* and *C*), which suggests that enhanced growth is at least in part mediated *via* histone methylation. We thus chose 10 μM EPZ-6438 for parallel SIRM experiments on A549 spheroids grown in Matrigel. We also employed a dual tracer approach, *i.e.* ^2^H_7_ (D_7_)-Glc + ^13^C_5_,^15^N_2_-Gln to expand the metabolic network coverage while eliminating potential sample batch effect with the single tracer approach.

**Figure 4:**
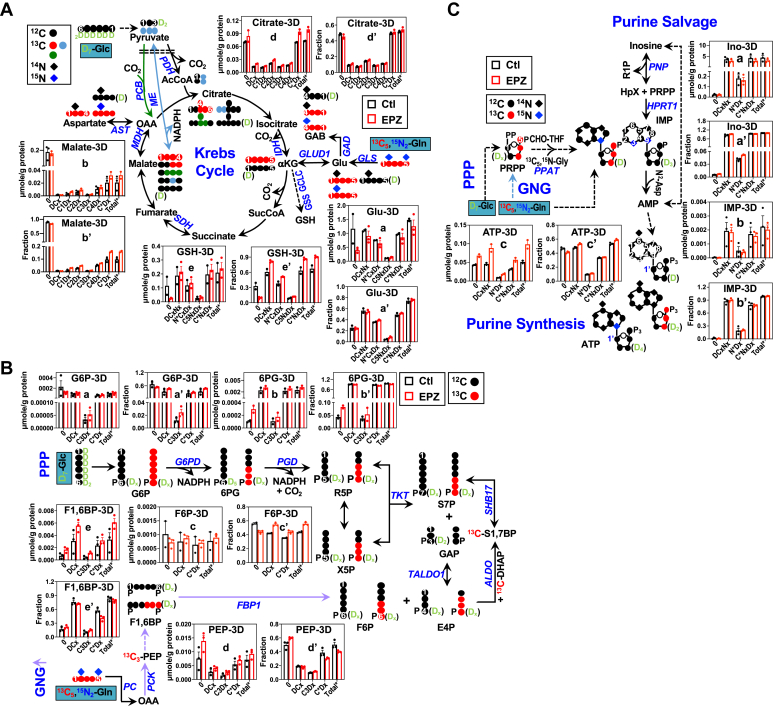
**EZH2 inhibition by EPZ-6438 activates the Krebs cycle and salvage synthesis of nucleotides in Matrigel-grown 3D A549 spheroids.** A549 spheroids treated with 10 µM EPZ-6438 in Matrigel were harvested, quenched, extracted, and analyzed by IC-UHR-FTMS, as described under Experimental Procedures. Metabolites in the Krebs cycle (*A*), GNG, PPP (*B*), and synthesis pathways of adenine nucleotides (*C*) were quantified to obtain µmole/g protein (a-e) and fractional enrichment (a’-e’) (n=2–3; each as biological replicate). The labeled IMP and ATP structures shown in *C* are consistent with the most abundant labeled isotopologs observed by IC-UHR-FTMS analysis, that is, D-, ^15^N_1_D_4_, and ^13^C_3_D_2_-ATP and D_3_^15^N_2_-, ^13^C_3_^15^N_1_D_1_-IMP. S1,7BP, sedoheptulose-1,7-bisphsphatate; GLS, glutaminase; PCK1, phosphoenolpyruvate carboxykinase; FBP1, fructose-bisphosphatase 1; SHB17, sedoheptulose-1,7-bisphatase; ALDO, aldolase, fructose-bisphosphate. , , ,: ^13^C; : ^15^N; D: ^2^H; C∗: ^13^C; Dx, N∗: ^15^N; Dx, Cx, Nx: 0-x number of D, ^13^C, ^15^N; Total∗, sum of all labeled. All other symbols, abbreviations, and annotations are as in Figure 1, Figure 2, Figure 3. See Table S4 for the statistics of changes in metabolites and their isotopologs. EZH2, Enhancer of Zeste Homolog 2; GNG, gluconeogenesis.

D_7_-Glc is converted to D_2_-pyruvate before entering the Krebs cycle, where D-labeled intermediates can co-transform with ^13^C_5_,^15^N_2_-Gln-derived ^13^C or ^13^C,^15^N-labeled intermediates to form doubly or triply labeled species such as ^13^C_5_,D_1_-citrate or ^13^C_2_,^15^N_1_,D_2_-Glu. Figure 4*A* showed the effect of EPZ-6438 treatment on D_7_-Glc and ^13^C_5_,^15^N_2_-Gln transformations *via* the Krebs cycle in A549 spheroids. EPZ-6438 had little effect on glycolysis as suggested by the lack of changes in the D-labeling patterns of glycolytic products (data not shown). Neither did EPZ-6438 impact Gln conversion to Glu *via* the glutaminase (GLS) reaction, as evidenced by the lack of change in the ^13^C_5_ labeling of Glu (C_5_N_x_D_x_, a-a’). However, EPZ-6438 altered the labeling patterns of subsequent Krebs cycle intermediates, malate (b’), citrate (d), and the anti-oxidant product GSH (e’). Most significantly, the level of D and ^13^C labeled citrate (C2Dx to C4Dx or ^13^C_2,3,4_-citrate) as well as sum of all labeled citrate (Total∗) (d) increased in response to EPZ-6438 while the corresponding fractional enrichment was not altered (d’). This points to enhanced turnover (both synthesis and utilization) of citrate *via* PDH- (•, ^13^C_4_-citrate as marker), ME- (•, ^13^C_2_-citrate as marker), and/or PC- (•^13^C_3_-citrate as marker)-initiated Krebs cycle reactions by EPZ-6438 (Fig. 4*A*). Enhanced utilization of products of PDH and ME reactions is consistent with increased fractional enrichment but not level of ^13^C_1_- and ^13^C_2_-malate (respective markers of ME and PDH-initiated reactions) in EPZ-6438-treated *versus* control A549 spheroids. This is also the case for GSH, as we saw increased fractional enrichment but not level of ^15^N-labeled (N∗CxDx), ^13^C-labeled (C∗NxDx), and sum of all labeled GSH (Total∗) in response to EPZ-6438 treatment. Enhanced utilization of anaplerotic ME products could fuel anabolic metabolism required for supporting the growth stimulation.

D_7_-Glc is also a substrate for PPP, where it provides D labeled R5P for fueling anabolic nucleotide synthesis, while ^13^C_5_,^15^N_2_-Gln is a carbon source for PPP only if it undergoes GNG transformations (Fig. 4*B*). We saw extensive ^13^C enrichment (C∗Dx) in PPP metabolites such as 6-phosphogluconate (6PG, b-b’), along with that in the glucogenic metabolites phosphoenolpyruvate (PEP, d-d’), F1,6BP (e-e’), and F6P (c-c’) (Fig. 4*B*). Interestingly, the ^13^C enrichment in 6PG was higher than that in its precursor G6P (a’); the latter is also produced from glycolysis. This result points to a preferred diversion of GNG-produced G6P to PPP in A549 spheroids. GNG activity was enhanced by EPZ-6438, as evidenced by the higher level and ^13^C enrichment of ^13^C_3_ labeled F1,6BP (C3Dx, e-e’), which is a good indicator of GNG. This presumably led to the higher enrichment of ^13^C_3_ labeled 6PG (b’) and G6P (a’) in EPZ-6438 *versus* control A549 spheroids. Reduced enrichment of ^13^C labeled F1,6BP (C∗Dx, e’) but enhanced enrichment of its product ^13^C labeled F6P (C∗Dx, c’) is also consistent with increased flow of GNG products to PPP (Fig. 4*B*).

To determine whether such enhanced GNG results in increased nucleotide synthesis, we tracked the ^13^C and D label incorporation into ATP *via* the purine nucleotide synthesis pathway, as shown in Figure 4*C*. We found an increase in the level (c) and enrichment (c’) of all isotopologues of ATP in EPZ-6438 *versus* control spheroids, although the level did not show statistical significance for some species. We also saw higher ^13^C (C∗NxDx) and ^15^N (N∗Dx) enrichment in inosine (a’) than that in IMP (b’) and ATP (c’), which suggests that inosine serves as a precursor, instead of product, of IMP and ATP synthesis. This is consistent with the prevalence of the salvage pathway in supporting ATP synthesis, as observed for A549 spheroids in the ^13^C_6_-Glc-based SIRM experiment (cf. Fig. 3*B*). Enhanced salvage synthesis by EPZ-6438 is also akin to that by EZH2 KD, which suggests that reprogrammed ATP synthesis was at least in part mediated by histone methylation in A549 spheroids.

As we showed above that EZH2 modulation of cancer cell metabolism depended on the presence of ECM, we then asked whether the nature of the ECM also impacts metabolic modulation. We grew A549 spheroids in Aggrewell plates, which enabled spheroid formation by allowing A549 cells to produce ECM without the need for Matrigel (Fig. S2*B*). The ECM generated by the human cells should approximate the human TME better than Matrigel, which is of mouse origin and varies within a single batch and between batches (27). The latter could be related to the different extent of growth stimulation by EPZ-6438 in 3 separate experiments (Fig. S2, *C*–*E*). In addition, as spheroids were isolated from each other in microwells, they did not form larger coalesced structures (cf. Fig. S1*D*), which can affect nutrient/O_2_ uptake and waste product release. We confirmed that matrix-free A549 spheroids produced their own ECM components such as collagens (COL1A1, COL3A1) and fibronectin (FN1), as shown in Fig. S3.

**Figure 5:**
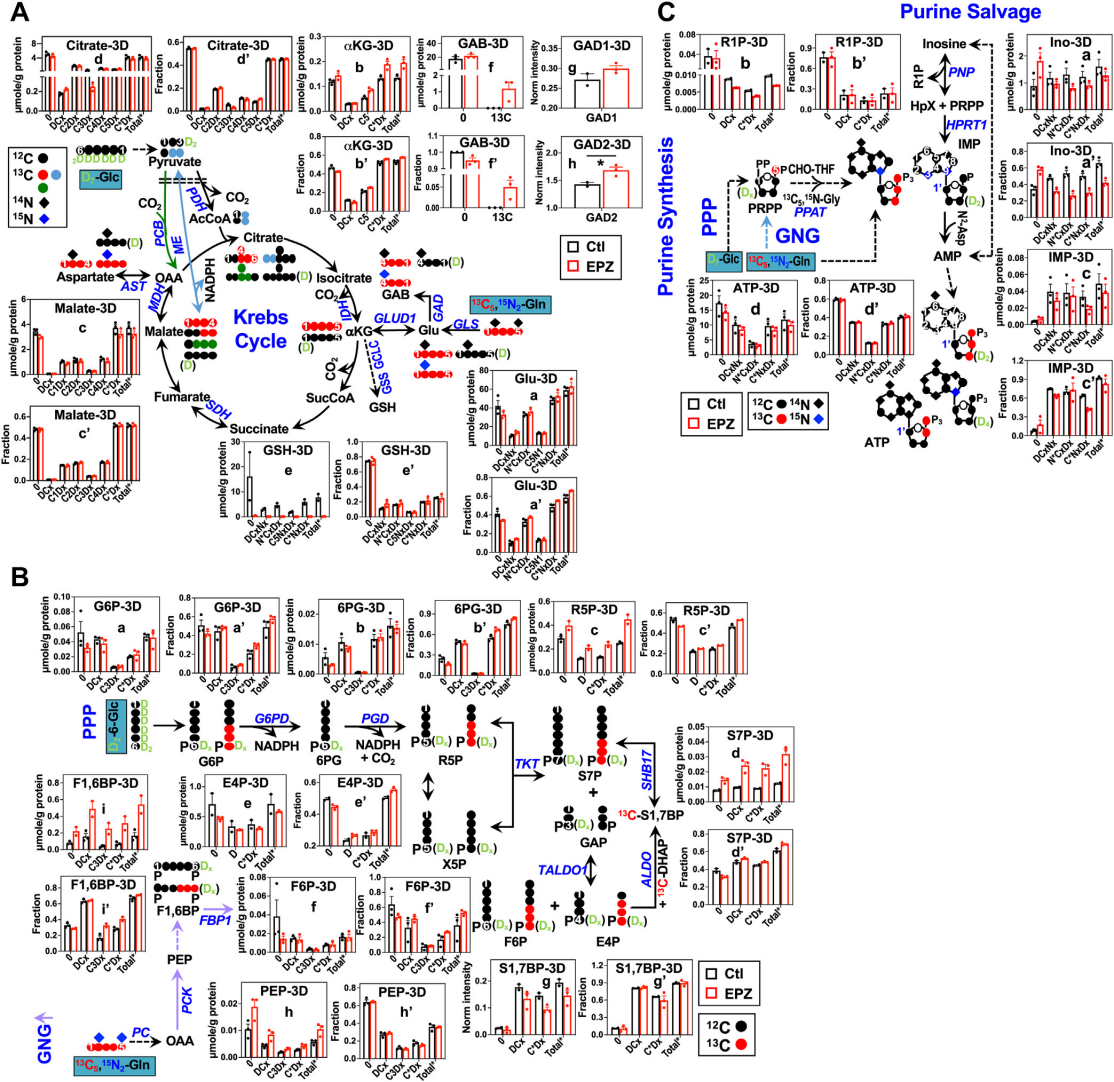
**EZH2 inhibition by EPZ-6438 activates GAB synthesis, gluconeogenesis, and PPP but blocks salvage synthesis of nucleotides in Matrigel-free 3D A549 spheroids.** Matrix-free A549 spheroids were cultured in an Aggrewell plate, treated with 10 µM EPZ-6438, harvested, and extracted, as described under Experimental Procedures. Metabolites in the Krebs cycle (*A*), GNG, PPP (*B*), and synthesis pathways of adenine nucleotides (*C*) were analyzed by IC-UHR-FTMS as in Figure 4 and by ^1^H NMR (f-f’) to obtain µmole/g protein and fractional enrichment (n = 3; each as biological replicate). The labeled IMP and ATP structures shown in (*C*) are consistent with the most abundant labeled isotopologs observed by IC-UHR-FTMS analysis, that is, ^15^N_1_D_4_, ^13^C_2_-, and ^13^C_3_D_2_- ATP; ^15^N_2_D_2_- and ^13^C_3_^15^N_1_-IMP. GAD1/2 protein expression (Ag-h) was analyzed by RPPA, as described in Experimental procedures. All symbols, abbreviations, and annotations are as in Figure 1, Figure 2, Figure 3. See Table S5 for the statistics of changes in metabolites and their isotopologs. EZH2, Enhancer of Zeste Homolog 2; GAB, γ-aminobutyrate; GNG, gluconeogenesis; PPP, pentose phosphate pathway.

We performed the same SIRM experiments on these isolated spheroids as those performed on Matrigel cultures in Figure 4. We saw more extensive incorporation of ^13^C from the Gln tracer into metabolites of the latter half of the Krebs cycle such as malate for isolated spheroids (c’, Fig. 5*A*) than for coalesced Matrigel-grown spheroids (b’, Fig. 4*A*). We also observed higher levels of Krebs cycle metabolites (citrate, αKG, malate) for isolated than for coalesced spheroids (Figs. 4*A* and 5*A* and data not shown). EPZ-6438 treatment enhanced the buildup and enrichment of ^13^C labeled (C∗Dx) αKG in isolated (b-b’, Fig. 5*A*) but not in coalesced spheroids (data not shown). Notably, there were minimal or no changes in malate/citrate labeling (c-d, c’-d’) and attenuated ^13^C incorporation into GSH (e) in isolated spheroids (Fig. 5, A) contrasting with enhanced ^13^C enrichment of the counterparts in coalesced spheroids (e’, Fig. 4*A*). Moreover, ^13^C-GAB production from ^13^C_5,_^15^N_2_-Gln was enhanced by EPZ6-438 (f-f’, Fig. 5*A*), which was similarly observed for ^12^C-GAB in A6-transformed EZH2 KD spheroids (Fig. 1*A*-h’). Consistently, GAD2 protein expression was elevated (h, Fig. 5*A*) in response to EPZ-6438, as the case for the A6 spheroids (k, Fig. 1*A*). The GAB level in coalesced spheroids was below NMR detection. Thus, EZH2-reprogrammed GAB metabolism could be mediated by the PRC2-dependent process, but this event depends on the nature of ECM required for spheroid formation.

We further observed extensive ^13^C labeling of the GNG products PEP (h-h’) and F1,6BP (i-i’) as well as PPP metabolites including 6PG, S7P, E4P, and F6P (b-f, b’-f’) (Fig. 5*B*), which must have derived from ^13^C-Gln. As in the coalesced spheroids, the ^13^C enrichment of 6PG (b’) was higher than that of G6P (a’) in isolated spheroids, which again points to preferred diversion of GNG carbons to PPP, rather than to glucose production. We also saw high ^13^C enrichment of an unusual metabolite, sedoheptulose-1,7-bisphosphate (S1,7BP), which has been shown to be synthesized from F6P and E4P *via* aldolase (ALDO) reactions to produce S7P, thereby replenishing R5P during high growth demand in yeast (28). The higher ^13^C enrichment of S1,7BP (g’) than that of S7P (d’) is consistent with the occurrence of this pathway in isolated A549 spheroids. As the case for A6 spheroids, EPZ-6438 enhanced the buildup and enrichment of D and ^13^C labeled R5P (c-c’) and S7P (d-d’) but not the buildup of their precursors (D and ^13^C labeled S1,7BP, E4P, F6P, and G6P) (e-g, a). In addition, both level (i) and enrichment (i’) of ^13^C labeled F1,6BP (C3Dx and C∗Dx) increased in response to EPZ-6438, which point to enhanced synthesis of F1,6BP *via* GNG and downstream utilization such as *via* the PPP to support R5P production. EPZ-6438 activation of GNG is also consistent with the enhanced buildup of ^13^C labeled PEP (h). Moreover, there was evidence for increased transformation of glycolytic products (*e.g.* D-labeled PEP and F1,6BP) *via* the PPP to support the buildup of D-labeled S7P and R5P (DCx, Fig. 5*B*). Altogether, these data revealed that PRC2-dependent process could govern GNG and PPP activation in isolated A549 spheroids with self-generated ECM and that such modulation in coalesced spheroids in Matrigel was less evident.

However, in contrast to the case for coalesced spheroids (Fig. 4*C*), we did not see enhanced buildup or enrichment of D and ^13^C labeled ATP (DCxNx and C∗NxDx; d-d’, Fig. 5*C*) in isolated spheroids, despite enhanced PPP. This lack of changes in ATP synthesis could be reflected by reduced D and ^13^C labeling of inosine (a’), R1P (b’, salvage product of inosine), and IMP (c’), which could underlie the attenuated salvage synthesis of ATP, thereby nullifying the effect of activated PPP on nucleotide synthesis. Differential contribution of *de novo* and salvage synthesis pathways to ATP production could account for the occurrence of distinct abundant isotopologues for ATP (^15^N_1_D_4_, ^13^C_2_-, and ^13^C_3_D_2_- ATP) *versus* IMP (D_2_^15^N_2_-, ^13^C_3_^15^N_1_-IMP) in isolated spheroids (Fig. 5*C*). This was also the case for coalesced spheroids (Fig. 4*C*). Thus, although both isolated A549 spheroids with their own ECM and coalesced spheroids in Matrigel shared a high capacity for GNG and diversion of GNG products into nucleotide synthesis, their responses to EPZ-6438 differed in these anabolic activities. Lack of changes in ATP synthesis is consistent with minimal growth stimulation (Fig. S2*B*) induced by EPZ-6428 in isolated spheroids *versus* enhanced ATP synthesis and growth stimulation elicited by EPZ-6428 in coalesced spheroids (Fig. S2, *A* and *C*).

## Discussion

EZH2 is widely overexpressed in human lung cancer tissues (7) and cells (6), and its suppression inhibits 2D lung cancer cell growth as shown here and by others (6). However, *EZH2* suppression had an opposite effect on the 3D spheroid growth of lung adenocarcinoma A549 cells. This highlights the crucial importance of cell-cell and/or cell-matrix interactions on modulating EZH2’s function in A549 cells, which is previously unknown. We also learned that the growth stimulation induced by *EZH2* suppression commensurate with enhanced anaplerotic pyruvate carboxylation and PPP, needed for fueling growth-requiring anabolic metabolism by replenishing Krebs cycle intermediates and supporting nucleotide synthesis, respectively. We further found activation of GAB synthesis pathway by EZH2 KD in A549 spheroids, most likely mediated *via* GAD1/2 overexpression. This pathway can in turn activate GAB signaling in the TME (22) and its absence in 2D cells could be related to the lack of demand for cell-cell and/or cell-matrix interactions. GAB synthesis can also serve to fuel energy metabolism in the Krebs cycle (23), which is consistent with the growth stimulation induced by EZH2 KD (Fig. S1*B*). It is interesting to note that unlike mouse regulatory T cells (23), Glc or Gln did not support GAB synthesis in A549 spheroids, because neither tracer led to high fractional enrichment of ^13^C labeled GAB (Fig. 5 and data not shown). Of another note, the two *shEZH2* vectors had different effects on A549 growth and metabolism. By relating to the growth and metabolic effects induced by EPZ-6438 (a specific inhibitor of EZH2 enzyme activity), the A6 short-hairpin sequence appears to be more effective than the A9 short-hairpin sequence at *EZH2* suppression.

The dual tracer/triple isotope approach using D_7_-Glc simultaneously with ^13^C_5_,^15^N_2_-Gln enabled us to expand both the range and specificity of metabolic network tracing, *e.g.* simultaneous tracing of GNG, PPP, and glycolysis. Using ^13^C_6_-Glc alone, we could not confirm the contribution of GNG to ^13^C scrambling of the PPP metabolites in 3D A549 spheroids (Fig. 2*B*). However, this was confirmed by tracking ^13^C_5_,^15^N_2_-Gln transformations into PEP, F1,6BP, and the sugar phosphate metabolites of PPP (Figs. 4 and 5). In turn, we uncovered activation of this pathway in 3D spheroids by EPZ-6438 and its dependence on the nature of the ECM, i.e. mouse *versus* human origin. We also discovered preferential diversion of GNG products for fueling nucleotide synthesis largely *via* the salvage pathway. Reliance on GNG, instead of glucose, for neoribogenesis and nucleotide synthesis could offer growth and survival advantages for cancer cells when glucose is depleted in the TME (29, 30), which occurs readily during cancer cell expansion.

Moreover, by comparing reprogrammed metabolism elicited by *EZH2* KD *versus* EPZ-6438, we surmised that activation of the Krebs cycle, PPP, GAB synthesis, GNG, and salvage synthesis of ATP is modulated by PRC2 or H3K27me3-dependent mechanism while glycolytic inhibition was mediated by PRC2-independent processes. The EZH2 modulated metabolic activation can be targeted to synergize with EPZ-6438 in anti-cancer action. We also uncovered the dependence of EZH2 modulation of GNG, PPP, and salvage synthesis of ATP on ECM properties. Further studies will be necessary to elucidate the regulatory pathways involved in EZH2 modulation of central metabolism and its dependence on ECM properties. Suffice to say, the single use of EZH2 inhibitors in treating lung cancer patients may not be effective based on our findings that EZH2 inhibition failed to block A549 spheroid growth.

In conclusion, we found EZH2’s modulation of A549 cell growth and growth-related central metabolic networks to depend highly on the presence of cell-cell and/or cell-matrix interactions. In particular, *EZH2* suppression elicited PPP, GAB production, and salvage synthesis of ATP in 3D spheroids *versus* 2D cells, which is consistent with its growth-stimulating effect in 3D spheroids. We also saw extensive Gln transformations *via* GNG and preferred diversion of the GNG products into the PPP and nucleotide synthesis in 3D spheroids. GAB synthesis, GNG, PPP, and salvage synthesis of purine nucleotides were activated *via* a PRC2-dependent mechanism, but such activation varied for A549 spheroids formed in mouse Matrigel or self-produced human ECM. Thus, our findings highlight the importance of the presence and nature of ECM in studying the function of EZH2 and its inhibitors’ effects in cancer cells for modeling the *in vivo* outcomes.

## Experimental procedures

### *EZH2* suppression

Vectors used in this study included the pLKO.1 lentiviral backbone with *shGFP* (3′- GCCC(GCAAGCTGACCCTGAAGTTCAT)TCAAGAG(ATGAACTTCAGGGTCAGCTTGC)TTTT -5′), *shEZH2*-A6 (3′ -CCGG-TATTGCCTTCTCACCAGCTGC-CTCGAG-GCAGCTGGTGAGAAGGCAATA-TTTTTG -5′) or *shEZH2*-A9 (3′- CCGG-CGGAAATCTTAAACCAAGAAT-CTCGAG-ATTCTTGGTTTAAGATTTCCG-TTTTT-5′) sequences cloned after the U6 promoter. The *shGFP* vector is available on Addgene (#12273) (31), and *shEZH2* vectors were purchased through Sigma (TRCN0000040076 and TRCN0000040073) and previously validated (17, 32). Lentivirus was packaged using the above vectors transfected with VSVG and vpr-delta-8.2 vectors into HEK293T cells with FuGENE 6 (Promega) using established protocols (33). After 48 h, culture supernatants were collected, filtered with 0.45 μm filters and either stored in aliquots in −80 °C or applied directly to target cells. After transduction, cultures were selected for lentiviral integration with 1 μg/ml puromycin (Sigma #P9620).

### 2D and 3D cell culturing

A549 cells were purchased from ATCC (catalog number CRM-CCL-185) and certified as free of *mycoplasma* contamination. The derivative lines of A549 were made in the presence of plasmocin to limit the possibility of *mycoplasma* contamination. For 2D culturing, A549 cells transduced with shGFP, A6, or A9 virus were grown on 10- or 15-cm plates in Dulbecco’s modified Eagle’s medium (DMEM) base medium (without Glc and Gln) supplemented with 2 g/L (11.1 mM) Glc, 2 mM Gln, 10% fetal bovine serum (FBS), 100 U/ml penicillin, 100 μg/ml streptomycin (full DMEM), and 1 μg/ml puromycin to 90% confluency. Cells were then detached with 1× TrypLE (ThermoFisher) and seeded onto 384-well plates for viability testing or 6-well plates for ^13^C_6_-Glc tracer experiment as described below.

For 3D culturing in LunaGel (Gelomics), detached cells were suspended in LunaGel stock and mixed with the photoinitiator (dissolved in PBS) in 1:1 ratio before they were seeded into 24-well plates (500,000 cells in 0.25 ml/well) and illuminated under 400 nm light for 1 min to solidify the gel, as per vendor’s protocol. Cells were then cultured in spheroid medium (SM) composed of DMEM/F12 (Fisher Scientific, #11320033) + 10% FBS (Atlanta Biologicals, # S11550) + 1X B27 (Fisher Scientific, #17–504–044) + 1X insulin-transferrin-selenium (ITS-G, catalog number 41400–045, ThermoFisher) + 1× Glutamax supplement (ThermoFisher Scientific, Cat No. 35050061) + 1X Anti-Anti (Gibco, # 15240–062) + 1 μg/ml puromycin (Fisher Scientific, # mt61385RA) for 6 days of spheroid growth with half of the medium refreshed every other day before the ^13^C_6_-Glc tracer experiment as described below.

For 3D culturing in Matrigel (Corning; #47743–722), detached cells were suspended in SM and mixed with Matrigel stock in 1:1 ratio at 4 °C before they were seeded into 384- (20,000 cells in 10 μl/well) or 24-well plates (400,000 cells in 0.2 ml/well). Gel was solidified at 37 °C/5% CO_2_ for 20 min before 80 μl (384-well plate) or 1 ml (24-well plate) SM was added to each well. Cells were cultured in SM for 6 days of spheroid growth before treatment with 10 μM EPZ-6438 or vehicle (DMSO) for 10 days for the viability testing or dual Glc and Gln tracer experiments as described below. Half of the medium were refreshed every other day.

For 3D culturing without added matrix, detached cells were seeded at 300,000 cells/well in a 24-well Aggrewell 400 plate (StemCell Technologies), as per vendor’s protocol. Each well contains an array of microwells 400 μm in diameter to enable up to 1200 isolated spheroids to be formed. Briefly, Bio-float FLEX coating solution (400 μl, Facellitate #F202005) was added to each well and centrifuged at 1300*g* at 4 °C for 5 min to remove bubbles. The plate was kept for 5 min at room temperature for complete coating. Bio-float solution was aspirated, the plate was air-dried for ≥ 30 min to form low cell-retention surface in microwells, and each plate well was rinsed with 2 ml DMEM/F12 base medium. After removing the wash medium *via* vacuum suction, cells suspended in a lung organoid medium (LOM; see Table S1 for composition) were added to each well and mixed gently before spinning at 1000*g* at 4 °C for 1 min to evenly deposit cells into each microwell for spheroid formation. Spheroids in each plate were grown at 37 °C/5% CO_2_ with gentle rocking.

### Growth analysis

For 2D cultures, A549 cells were seeded in a 384-well at 600 to 900 cells/well and grown in the full DMEM medium + 1 μg/ml puromycin for 3 days before viability test using the PrestoBlue (PB) cell viability reagent (ThermoFisher) per vendor’s protocol. Briefly, 2X PB + 4 μg/ml Hoechst dye in SM (20 μl) was added to each well containing cells in 20 μl SM medium. The plate was incubated at 37 °C/5% CO_2_ for 30 min before reading using excitation/emission wavelengths of 560/590 nm for PB and 360/460 nm for Hoechst.

For 3D cultures in Matrigel, viability testing was performed similarly as that for 2D cultures except for the addition of 40 μl of the test reagent to spheroids in 30 μl SM medium + 10 μl Matrigel to make final 1X test reagent and incubation at 37 °C/5% CO_2_ for 1 h with gentle rocking to facilitate exchange.

### Invasion assay

The Transwell invasion assay was performed on 2D A549 cell cultures following Honk *et al*’s protocol (34) with minor modifications. Briefly, the Transwell insert (Falcon Cell Culture Inserts 353097 for 24-well chambers, 8 μm pore size) was coated with a mixture of Matrigel (Corning) and DMEM media (1:2, v/v) and solidified at 37 °C for 30 min. One hundred microliters of cell suspension (5 × 10^4^ cells) in DMEM with 1% FBS and 600 μl of the DMEM media with 10% FBS was then added on top of the gel and in the lower chamber, respectively. Cells were incubated at 37 °C and 5% CO_2_ for 24 h. After incubation, cells were fixed with 10% formalin and stained with 0.2% crystal violet in 10% formalin. Matrigel and uninvaded cells were removed with ice-cold water washes. The inserts were then dried at room temperature and the crystal violet was eluted with 10% glacial acetic acid before quantification at 590 nm.

The invasion study for the 3D spheroids were performed as described by Mahmoud *et al*. (35) with modifications. Briefly, cells were suspended in LOM media and seeded in a 384-well (instead of a 96-well) round bottom plate with low cell retention surface (Corning 4516) at 1000 cells in 80 μl/well. They were centrifuged at 1000*g* for 1 min before incubation at 37 °C and 5% CO_2_ with gentle rocking for 4 days to allow spheroid formation. Fifty microliters of media was aspirated from each well at day 4 before adding 30 μl of Matrigel to make 1:1 media:Matrigel. The plate was incubated at 37 °C for 30 min to solidify the gel before adding 30 μl/well of LOM media on top of the Matrigel. Invasion of the 3D spheroids into the gel was monitored for 6 days using an EVOS M7000 imaging system (Invitrogen). Spheroid areas were measured at day 0 to day 6 of Matrigel addition using ImageJ software version 1.53 (National Institutes of Health, https://imagej.net/ij/download.html). % invasion, I, was calculated as:

$$I=100\;x\left(A_{6}-A_{0}\right)/A_{0}$$

where A_0_ and A_6_ are the areas at day 0 and day 6, respectively.

### Cell migration assay

The 2D migration assay was done as described by Mo *et al*. (36) except for the use of a 96 well (instead of 24-well) plate. We seeded 4500 cells per well in the 96 well-plate. The scratches were performed at day 4 of the seeding, followed by monitoring healing for up to 26 h.

### Clonogenic assay

The clonogenic assay was conducted as described by Sarnella *et al*. (37) with minor modifications. For the seeding density, we used 1000 cells/well instead of 500 cells/well and the bound crystal violet was eluted with 10% glacial acetic acid instead of 25% methanol.

### Immunofluorescent staining of the A549 spheroids and confocal microscopy

A549 spheroids bearing shGFP, shA6, or shA9 virus were grown in LOM media for 14 days in a 384-well round bottom plate with low cell retention surface as described in the 3D invasion assay. Spheroids were transferred from each well to 2 ml tubes, fixed in 4% paraformaldehyde overnight at 4 °C and washed with PBS twice each at 20 min with nutation. They were then transferred individually into a well of 3D flowchips (Protein Fluidics Inc). IF staining of A549 spheroids was done with primary antibodies (see below) and Alexa 488-goat anti-rabbit or Alexa 647-goat anti-mouse secondary antibodies (Invitrogen A-11008, A-21235) using a microfluidic-based automated staining system (Pu.MA System, Protein Fluidics) per vendor’s protocol. Briefly, primary and antibodies were diluted 100-fold while secondary antibodies were diluted 1000-fold plus 0.5 μg/ml DAPI in a blocking buffer containing 5% goat serum (Invitrogen 50062Z) + 0.05% BSA + 0.2% Triton in PBS. Wash buffer (PBS), blocking buffer, and diluted antibodies (20 μl each) were added into the designated wells of the 3D flowchip without generating bubbles before the flowchips were placed into the Pu.MA System. IF staining was performed with the following program: 1 h permeabilization + blocking and 16/2 h of primary/secondary antibody incubation. At the end of staining, half of the PBS in each well was replaced with an anti-fade solution (Stay-brite, Ursa bioscience LLC) to minimize fluorescence quenching before imaging by confocal microscopy (Olympus FV-1000).

**Table:** 

Protein targets	Vendor	Catalog number	Dilution	Validation per vendor
COL1A1	Proteintech Group	67288-1-Ig	1:100	Single WB band
COL3A1	Proteintech Group	22734-1-AP	1:100	KO/KD responsive
FN1	Proteintech Group	66042-1-Ig	1:100	KO/KD responsive

### Stable isotope tracer experiments

For the ^13^C_6_-Glc experiment, cells were rinsed in the DMEM or DMEM/F12 base medium to remove unlabeled Glc and lactate before cell medium was changed to the full DMEM or SM medium with Glc replaced by 0.2% ^13^C_6_-Glc for 1 or 2 days for the 2D or 3D cultures, respectively. Two hundred microliters of medium were removed at the start and end of the experiments for extraction of polar metabolites in 80% cold (−80 °C) acetone for 30 min, as described previously (38) At harvest, 2D cells were rinsed 3 times each within 30 s in ice-cold PBS and then Nanopure water to remove medium salts before metabolic quenching in cold (−20 °C) acetonitrile and extraction for polar metabolites and proteins in acetonitrile:Nanopure water:chloroform in 2:1.5:1 ratio as described previously (39). 3D spheroids in LunaGel were harvested by cutting gel into small pieces before incubating in 10 mg/ml Collagenase I (catalog number SCR103, Sigma) at 0.2 ml/well and 37 °C/5% CO_2_ for 10 min with gentle rocking, rigorous pipetting to further break up the gel, and continued 37 °C incubation for another 5 min to achieve complete dissolution. To remove the gel and medium components, spheroids were transferred to a 15 ml conical tube by suspension in 2 × 1 ml cold PBS plus the addition of 10 ml cold PBS before pelleting at 4,650*g*/4 °C for 1 min. After aspirating the wash, pellets were washed again in 2 × 10 ml cold PBS before transferring to a 1.5 ml microfuge tube with 2 × 0.5 ml cold PBS and pelleting at 10,000*g*/4 °C for 1 min and aspirating the wash. Cell pellets were suspended quickly in 1 ml Nanopure water and pelleted at 15,000*g*/4 °C for 30 s to remove salts. After aspiration, pellets were metabolically quenched in cold (−20 °C) acetonitrile and extracted for polar metabolites and proteins as in the 2D cell experiment. 3D spheroids in Matrigel were similarly harvested and quenched except without the collagenase treatment but spheroids in Matrigel were suspended in cold PBS, let sit on ice for 10 min before transferring to a 15 ml tube, and washed 4 times with 10 ml cold PBS to remove Matrigel.

For the D_7_-Glc + ^13^C_5_,^15^N_2_-Gln tracer experiment, 2 days prior to the harvest, medium was changed to SM (for spheroids in Matrigel) or LOM (for spheroids without Matrigel) except that unlabeled Glc and Glutamax were replaced with 0.2% D_7_-Glc and 2 mM ^13^C_5_,^15^N_2_-Gln. Again, 200 μl medium was sampled at the start and end of the tracer treatment for extraction of polar metabolites as described above. At harvest, spheroids in Matrigel were suspended in 1 ml cold PBS, kept on ice for 10 min, and suspended again before transferring to a 15 ml conical tube with 2 x 1 ml cold PBS. Ten milliliter cold PBS was added to the tube before cell pelleting at 4,650*g*/4 °C for 1 min and aspirating the wash. This wash process was repeated 3 times to remove Matrigel. The final cell pellet was suspended and transferred in 2 x 0.75 ml cold PBS to a 2 ml microfuge tube and spun at 10,000*g*/4 °C for 1 min. Cell pellets were then rinsed quickly in Nanopure water and quenched in acetonitrile as described above. To harvest spheroids in AggreWell plates, the plate was kept on clean ice, and spheroids in each well were suspended in the spent media before transferring into a pre-tared 15 ml tube. The tubes were weighed and then centrifuged at 4650*g*/4 °C for 1 min before the cleared spent media were transferred into 2 ml tubes for further analysis. Each well was washed with 2 x 1 ml ice-cold PBS, and the washes were pooled with the spheroid pellet resuspended in 10 ml ice-cold PBS before pelleting at 4650*g*/4 °C for 1 min. The pellets were washed two more times with 10 ml each ice-cold PBS and pelleted each time at 4650*g*/4 °C for 1 min. The washed pellet was suspended in 2 x 0.65 ml ice-cold PBS, transferred to a 1.5 ml microfuge tube, and pelleted at 10,000×*g*/4 °C for 1 min. Next, spheroid pellets were rinsed quickly in Nanopure water to remove medium salts and then quenched in cold acetonitrile, as described above. The polar metabolites and proteins were extracted from the quenched spheroids similarly as described above for the 2D cell experiment, except that quenched spheroids were homogenized by pestling in a small volume (50–100 μl) of 60% acetonitrile before the addition of chloroform.

### NMR analysis

Lyophilized cell polar extracts were dissolved in 35 μl D2O (pD at 7.4) containing 17.5 nmoles of d6-DSS (2,2-dimethyl-2-silapentane-5-sulfonate, Cambridge Isotope Laboratories) for NMR analysis. NMR spectra were recorded at 15 °C on a Bruker AVANCE III NMR spectrometer at 16.45 T (Bruker Corp.) equipped with a 1.7 mm inverse triple resonance cryoprobe, as described previously (40). A ^1^H 90° pulse with solvent presaturation was used to acquire 1D ^1^H spectra with an 8403 Hz spectral width, 2 s acquisition time, 4 s relaxation delay, and 512 transients. The free induction decays were zero-filled to 131,072 points, apodized with a 1 Hz line-broadening exponential before Fourier-transformation, followed by phasing, baseline correction and referencing to the internal DSS resonance at 0 ppm. 1D ^1^H{^13^C} HSQC spectra were recorded with ^13^C broadband adiabatic decoupling during the acquisition time of 0.25 s. A total of 4200 data points were collected for each transient and 1024 transients were acquired with a 12 ppm (8403 Hz) spectral width and a recycle time of 2 s. The HSQC spectra were then apodized with an unshifted Gaussian function and 4 Hz exponential line broadening and zerofilled to 16,384 data points before Fourier transformation, phasing, and baseline correction. The metabolites were identified using in-house databases (41, 42, 43). The “peak picking” and line fitting routines in the MestReNova software (Mestrelab Research S.L., https://mestrelab.com) was used to quantify resonances of interest. Each peak area was calibrated to the methyl resonance of d6-DSS to determine the analyte quantity and then normalized to the cell protein content. The final metabolite content was expressed as μmole/g protein.

### IC-UHR-FTMS analysis

Polar extracts were analyzed using IC-UHR-FTMS operated in a data-independent MS/MS mode (44). All lyophilized cell extracts were reconstituted in 30 μl 18 MΩ water before analysis. The instrument used was a Dionex ICS-500+ ion chromatograph interfaced to a Thermo Scientific Orbitrap Fusion Tribrid Mass Spectrometer running on Orbitrap 3.3 control system with factory calibration achieving better than 0.3 ppm mass accuracy. All instrumental and data analysis procedures were as previously described (45).

### Western blotting and RPPA analysis

#### Western blot

Actively growing cultures were collected and lysed with RIPA buffer (0.5% deoxycholate, 1% IGEPAL-CA630, 0.1% sodium dodecyl sulfate, 150 mM NaCl, 50 mM Tris-8.1), cleared by centrifugation, and quantified for protein content using Pierce BCA Protein Assay Kit (Thermo Fisher Scientific, #23225). Hundred micrograms of protein extracts were prepared for SDS-PAGE by boiling with reducing agents, loaded in equal proportions for separation on 4 to 15% acrylamide gels (Bio-Rad, #456-1086), and transferred to nitrocellulose membranes (Cytiva, #10600002). Antibodies used for western blots were as follows: EZH2 (Cell Signaling, #5246s, 1:1000), H3K27me3 (Cell Signaling, #9733s, 1:1000), SUZ12 (Active Motif, #39357, 1:1000), EED (Millipore, #09-774, 1:1000). They were incubated with the membranes overnight at 4 °C. Histone H3 (AbCAM, Cat# ab1791, 1:5000) was used as loading control. After washing, membranes were incubated in secondary anti-rabbit IgG, HRP-linked antibody (Novus #NB7160, 1:10,000) for 1 h at room temperature. After washing, chemiluminescence was visualized with West Pico PLUS Chemiluminescent Substrate (Thermo Fisher Scientific, #34077) and exposure onto Amersham Hyperfilm ECL (Cytiva, #28-9068-36).

#### RPPA analysis

For reverse phase protein array (RPPA) assay, protein extracts (at 0.2–0.5 mg/ml) from the ^13^C_6_-Glc and dual tracer experiments were printed as two drops per spot onto a slide coated with 16 nitrocellulose membrane pads (Oncyte SuperNOVA 16 NC pads, Grace Bio-Labs) using a microarray printer (ArrayJet, Ltd) (46). Membrane pads on the slide were sealed with a ProChamber 16-well microarray system before protein staining using the Azure RPPA staining kit (#AC2233, VWR Scientific). The procedure involved blocking for 10 min in the blocking buffer and incubation in the staining solution for 5 min, followed by rinsing in the wash solution 4 times at 5 min each and in Nanopure water for 5 min. All steps were performed at room temperature with rotation at 200 rpm. Slides were then dried under vacuum and scanned at 700 nm emission wavelength using the InnoScan 710 AL Microarray Scanner (Innopsys, Inc) to quantify proteins deposited per sample spot. For immunostaining, slides were incubated in a Fluorescent Blot blocking buffer (#AC2190, Azure Biosystems) overnight at 4 °C with gentle rocking, followed by incubation in a primary antibody diluted in the blocking buffer against a target protein overnight at 4 °C with rocking, washing in a Blot washing buffer (#AC2113, Azure Biosystems), incubating with an anti-rabbit fluorescent secondary antibody (#AC2134, Azure Biosystems) at 1:8000 dilution in the blocking buffer for 1 h at room temperature, washing in the Fluorescent blot wash buffer (#AC2145, Azure Biosystems) with rotation at 200 rpm, and drying *via* vacuum suction. Slides were scanned at 800 nm emission wavelength with InnoScan 710 AL. Fluorescence image analysis of spots was done using Innopsys’s Mapix software (https://www.innopsys.com). Background fluorescence for each spot was subtracted from the fluorescence signal for that spot followed by normalization to the protein signal. Normalized signals were averaged across replicates (n = 3). The sources of the primary antibodies used are listed below.

**Table:** 

Protein targets	Vendor	Catalog number	Dilution	Validation per vendor
GAD1	Proteintech Group	10408-1-AP	1:100	KO/KD responsive
GAD2	Proteintech Group	20746-1-AP	1:100	Single WB band
TKT	Proteintech Group	11039-1-AP	1:100	KO/KD responsive
TALDO1	Proteintech Group	12376-1-AP	1:100	KO/KD responsive

## Data availability

All original data were stored in CESB’s computers and can be shared upon e-mail request to Teresa Fan

## Supporting information

This article contains supporting information: Figures S1–S3 and Tables S1–S5.

## Conflict of interest

The authors declare that they have no conflicts of interest with the contents of this article.
