# Supplementary material for: Metabolic reprogramming driven by EZH2 inhibition depends on cell–matrix interactions
Source: J Biol Chem. 2023 Nov 20;300(1):105485. doi: 10.1016/j.jbc.2023.105485 (PMC10770523; doi:10.1016/j.jbc.2023.105485)
Supplement: Supporting information [file mmc1.docx]

**Supporting information**

**Title: Metabolic reprogramming driven by EZH2 inhibition depends on cell-matrix interactions**

Teresa W-M Fan, Jahid M. Islam, Richard M. Higashi, Penghui Lin, Christine F. Brainson, and Andrew N. Lane

**Materials included: Figures S1-S3**; **Table S1-S5**

**Figure S1.**

**C**

**A**

**B**

**C**

**D**

**E**

**F**

**G**

**H**

**Figure S1. *EZH2* knockdown leads to opposite effects on the growth of A549 cells grown as 2D cultures or 3D spheroids.**

Two sh vectors (A6 and A9) or a control sh vector (shGFP) were used to suppress or maintain *EZH2* expression as described in **Experimental**. Suppression of EZH2 protein was confirmed by Western blotting in **A**, which also verified the expected consequence of this suppression in blocking H3K27me3.

shGFP, A6, and A9 cells were grown in 384-well plates as 2D cultures for 3 days or as 3D cultures in Matrigel for 6 days to measure viability by PresoBlue (**B** n=16; **C** n=6-7) (**B**). Cells were grown in 96-well plates for 4 days as 2D cultures for migration (scratch assay) (**D**; n=4-5). Cells were grown in 6-well plates for 9 days for colony formation (**E**; n=3). Cells were grown in 24-well as 2D cultures for 24 hr for invasion test (**F**; n=3). Cells were grown in 384-well plates as matrix-free spheroids before Matrigel addition (Day 0) and then for another 6 days (Day 6) for spheroid migration/invasion into Matrigel (**G**; n=6-8). Also illustrated in **G** are the spheroid images for shGFP cells at Day 0 and Day 6 of the invasion test from which the spheroid areas were acquired and used for calculating % Invasion as described in Experimental. A6 and A9 cells exhibited gross spheroid structures distinct from that of GFP cells when grown in Matrigel (4x objective) (**H**). All replicates were biological replicates. *, **, ***, *****: p-value ≤ 0.05, 0.01, 0.005, 0.0005, respectively; ns: not significant (by T-test).

**Figure S2.**

**B**

**A**

**E**

**D**

**C**

**Figure S2. EZH2 inhibition leads to growth stimulation in A549 spheroids but the extent of this stimulation is matrix dependent.**

A549 cells were grown as 3D spheroids in Matrigel and treated with different doses of a specific EZH2 inhibitor EPZ-6438 (EPZ) as described in **Experimental Procedures**. Growth was measured by the of PrestoBlue (PB) fluorescence normalized against NucBlue fluorescence (N) in **A** (n=8). A549 cells were grown in a 24-well Aggrewell plate to form isolated spheroids without added matrix, as shown in **B**, and growth was measured by protein weight (n=3). In **C**-**E**, A549 spheroids in 24-well Transwell^TM^ plates were exposed to 10 µM EPZ in Matrigel for 10 days in 3 separate experiments and growth was measured by protein weight (n=2-3). All replicates were biological replicates.

**Figure S3.**

**Figure S3. Matrix-free A549 spheroids produce ECM components.**

A549 cells harboring shGFP, shA6, and shA9 vectors were grown as 3D spheroids in round bottom 384-well plate with low cell retention surface for 6 days. Spheroids were fixed in 4% paraformaldehyde and immunostained for COL1A1, COL3A1, FN1, and nuclei (with DAPI) in a Pu.Ma system 3D (Protein Fluidics) as described in **Experimental Procedures**. Spheroid images were acquired on an FV-1000 Olympus Confocal microscope with an 20X (FN1) or 40X (COL1A1, COL3A1) objective.

**Table S1. Composition of Lung Organoid Medium (LOM)**

| **Component** | **Final conc.** | **unit** |
| --- | --- | --- |
| DMEM/F12 (Fisher Scientific, Cat No. 11320033) |  |  |
| FBS (Atlanta Biologicals, Cat No. S11550) | 2 | % |
| FGF 7 (PeproTech, Cat No. 100-19) | 20 | ng/ml |
| FGF 10 (PeproTech, Cat No. 100-26) | 50 | ng/ml |
| Noggin (PeproTech, Cat No. 120-10C) | 40 | ng/ml |
| A83-01 (R&D Systems, Cat No. 2939-CA-010) | 500 | nM |
| Y-27632 (Selleck Chemicals, Cat No. S1049) | 5 | µM |
| SB202190 (Sigma-Aldrich, Cat No. S7067-5MG) | 500 | nM |
| B27 supplement (Fisher Scientific, Cat No. 17-504-044) | 1x |  |
| N-Acetylcysteine (Sigma Aldrich, Cat No. A9165-25G) | 1.25 | mM |
| Nicotinamide (Sigma, Cat No. N0636-100G) | 5 | mM |
| GlutaMax 100x (ThermoFisher Scientific, Cat No. 35050061) | 1x |  |
| Anti-anti (100x) (Gibco, Cat No. 15240-062) | 1x |  |
| Primocin (Invivogen, Cat No. ant-pm-2) | 50 | µg/ml |

**Table S2. Student’s t test of metabolite isotopologues in control versus EZH2 knockdown 2D A549 cells in Figs. 1-3** ^1^

|  | p-value (µmole/g protein) ^2^ | | | | p-value (fraction) ^3^ | | | | |
| --- | --- | --- | --- | --- | --- | --- | --- | --- | --- |
| **ATP** | **0** | **5** |  |  | **0** | **5** | **Base** |  |  |
| GFP vs A6 | 6.37E-01 | 2.28E-01 |  |  | 2.40E-04 | 3.07E-04 | 2.66E-03 |  |  |
| GFP vs A9 | 8.03E-03 | 2.40E-03 |  |  | 2.91E-04 | 3.58E-04 | 3.87E-04 |  |  |
| A6 vs A9 | 2.31E-01 | 4.32E-01 |  |  | 8.74E-03 | 4.19E-02 | 1.69E-02 |  |  |
| **Asp** | **0** |  |  |  | **0** | **2** | **3** | **4** |  |
| GFP vs A6 | 2.09E-01 |  |  |  | 6.78E-03 | 1.93E-02 | 1.33E-02 | 5.15E-04 |  |
| GFP vs A9 | 1.95E-02 |  |  |  | 4.86E-03 | 1.34E-02 | 1.96E-03 | 2.48E-02 |  |
| A6 vs A9 | 6.16E-01 |  |  |  | 1.74E-01 | 4.51E-01 | 2.28E-02 | 3.07E-01 |  |
| **F6P** | **6** |  |  |  | **Scr** |  |  |  |  |
| GFP vs A6 | 2.04E-01 |  |  |  | 5.00E-02 |  |  |  |  |
| GFP vs A9 | 4.52E-04 |  |  |  | 1.80E-01 |  |  |  |  |
| A6 vs A9 | 4.47E-02 |  |  |  | 2.38E-01 |  |  |  |  |
| **Fumarate** | **0** | **2** | **3** | **4** | **0** | **2** | **3** | **4** |  |
| GFP vs A6 | 4.21E-01 | 4.38E-01 | 5.97E-01 | 8.26E-02 | 3.62E-03 | 1.60E-02 | 2.63E-03 | 1.23E-01 |  |
| GFP vs A9 | 1.94E-02 | 3.72E-01 | 6.95E-02 | 3.35E-01 | 1.57E-02 | 5.47E-02 | 1.71E-03 | 1.01E-02 |  |
| A6 vs A9 | 1.38E-01 | 2.09E-01 | 1.75E-01 | 1.34E-01 | 5.47E-01 | 7.91E-01 | 5.50E-01 | 9.54E-01 |  |
| **G6P** | **6** |  |  |  | **Scr** |  |  |  |  |
| GFP vs A6 | 1.86E-01 |  |  |  | 4.73E-01 |  |  |  |  |
| GFP vs A9 | 1.06E-02 |  |  |  | 1.69E-03 |  |  |  |  |
| A6 vs A9 | 7.59E-02 |  |  |  | 4.95E-01 |  |  |  |  |
| **Glu** | **0** | **2** | **3** | **4** | **0** | **2** | **3** | **4** |  |
| GFP vs A6 | 1.45E-01 | 2.46E-01 | 7.62E-01 | 5.84E-01 | 5.59E-04 | 6.35E-03 | 4.88E-02 | 1.64E-05 |  |
| GFP vs A9 | 1.39E-03 | 2.52E-03 | 2.16E-01 | 4.90E-03 | 1.21E-03 | 6.95E-03 | 7.30E-03 | 3.81E-05 |  |
| A6 vs A9 | 1.91E-01 | 2.75E-01 | 4.81E-01 | 1.95E-01 | 1.79E-01 | 2.79E-01 | 3.77E-01 | 2.08E-01 |  |
| **GSH** | **0** | **1** | **2** |  | **0** | **1** | **2** | **3** | **4** |
| GFP vs A6 | 2.92E-01 | 1.94E-02 | 6.59E-01 |  | 4.45E-04 | 1.76E-03 | 9.09E-04 | 1.69E-04 | 8.34E-05 |
| GFP vs A9 | 1.46E-03 | 3.07E-02 | 1.82E-02 |  | 1.10E-03 | 6.80E-03 | 1.23E-03 | 1.68E-03 | 1.01E-03 |
| A6 vs A9 | 2.40E-01 | 4.95E-01 | 3.68E-01 |  | 2.95E-01 | 4.95E-01 | 1.20E-01 | 7.84E-02 | 9.82E-02 |
| **Inosine** | **0** | **Base** |  |  | **0** | **Base** |  |  |  |
| GFP vs A6 | 8.70E-02 | 2.66E-02 |  |  | 3.68E-03 | 4.34E-02 |  |  |  |
| GFP vs A9 | 4.86E-02 | 1.91E-02 |  |  | 5.23E-03 | 5.94E-01 |  |  |  |
| A6 vs A9 | 7.89E-03 | 1.60E-01 |  |  | 5.64E-01 | 4.40E-01 |  |  |  |
| **Lactate** | **0** | **2** | **3** |  | **2** | **Succinate** | **0** | **2** | **3** |
| GFP vs A6 | 1.27E-01 | 5.96E-01 | 4.87E-01 |  | 6.49E-03 | GFP vs A6 | 2.55E-02 | 4.91E-02 | 1.04E-02 |
| GFP vs A9 | 2.51E-02 | 1.47E-03 | 1.04E-03 |  | 9.33E-02 | GFP vs A9 | 2.18E-01 | 6.23E-02 | 2.16E-01 |
| A6 vs A9 | 5.77E-02 | 3.08E-02 | 3.72E-02 |  | 2.82E-01 | A6 vs A9 | 4.44E-02 | 4.25E-02 | 6.48E-02 |
| **Malate** | **1** |  |  |  | **0** | **1** | **2** | **3** | **4** |
| GFP vs A6 | 2.09E-01 |  |  |  | 1.84E-03 | 1.87E-02 | 5.44E-03 | 4.78E-03 | 5.21E-04 |
| GFP vs A9 | 4.20E-02 |  |  |  | 6.85E-03 | 3.05E-02 | 1.55E-02 | 1.89E-03 | 1.07E-02 |
| A6 vs A9 | 4.95E-01 |  |  |  | 3.75E-01 | 4.95E-01 | 8.49E-01 | 6.53E-02 | 2.04E-02 |
| **R5P** | **5** | **Scr** |  |  | **0** | **5** |  |  |  |
| GFP vs A6 | 2.61E-01 | 3.86E-01 |  |  | 3.85E-02 | 2.28E-02 |  |  |  |
| GFP vs A9 | 1.93E-02 | 3.19E-02 |  |  | 1.13E-01 | 6.95E-02 |  |  |  |
| A6 vs A9 | 1.59E-01 | 7.57E-01 |  |  | 6.28E-01 | 8.75E-01 |  |  |  |
| **S7P** | **7** | **Scr** |  |  | **7** | **Scr** |  |  |  |
| GFP vs A6 | 4.10E-01 | 3.58E-02 |  |  | 3.02E-02 | 2.43E-02 |  |  |  |
| GFP vs A9 | 2.29E-04 | 2.20E-01 |  |  | 7.77E-02 | 7.46E-02 |  |  |  |
| A6 vs A9 | 7.60E-02 | 4.70E-02 |  |  | 8.83E-01 | 6.55E-01 |  |  |  |
| **Citrate** | **0** | **2** | **3** | **4** |  |  |  |  |  |
| GFP vs A6 | 5.34E-01 | 3.96E-01 | 9.83E-01 | 6.80E-01 |  |  |  |  |  |
| GFP vs A9 | 1.13E-01 | 2.49E-03 | 7.75E-01 | 9.85E-03 |  |  |  |  |  |
| A6 vs A9 | 5.95E-01 | 1.93E-01 | 8.45E-01 | 3.22E-01 |  |  |  |  |  |
| **6PG** | **6** | **Scr** |  |  |  |  |  |  |  |
| GFP vs A6 | 2.32E-01 | 1.95E-01 |  |  |  |  |  |  |  |
| GFP vs A9 | 1.11E-03 | 3.49E-03 |  |  |  |  |  |  |  |
| A6 vs A9 | 1.43E-01 | 3.61E-02 |  |  |  |  |  |  |  |
| **R1P** | **5** |  |  |  |  |  |  |  |  |
| GFP vs A6 | 2.85E-01 |  |  |  |  |  |  |  |  |
| GFP vs A9 | 1.44E-02 |  |  |  |  |  |  |  |  |
| A6 vs A9 | 6.90E-01 |  |  |  |  |  |  |  |  |
|  | *p*-value (mmole/g protein) | |  |  |  |  |  |  |  |
| **Lactate_med_** | **3-^13^C** |  |  |  |  |  |  |  |  |
| GFP vs A6 | 3.01E-01 |  |  |  |  |  |  |  |  |
| GFP vs A9 | 4.89E-02 |  |  |  |  |  |  |  |  |
| A6 vs A9 | 2.56E-01 |  |  |  |  |  |  |  |  |
| **Glc_med_** | **1-^13^C** |  |  |  |  |  |  |  |  |
| GFP vs A6 | 5.97E-01 |  |  |  |  |  |  |  |  |
| GFP vs A9 | 7.26E-02 |  |  |  |  |  |  |  |  |
| A6 vs A9 | 6.02E-02 |  |  |  |  |  |  |  |  |

^1^ ^13^C isotopologues of metabolites listed are as in **Figs. 1**-**3**.

^2^ *p*-values for metabolite amounts normalized to protein mass

^3^ *p*-values for fractional enrichment of unlabeled and ^13^C-labeled metabolites.

**Table S3. Student’s t test of metabolite isotopologues in control versus EZH2 knockdown A549 spheroids in Figs. 1-3** ^1^

|  | *p*-value (µmole/g protein) ^2^ | | | | | *p*-value (fraction) ^2^ | | | | |
| --- | --- | --- | --- | --- | --- | --- | --- | --- | --- | --- |
| **Asp** | **0** | **1** | **2** | **3** | **4** | **0** | **1** | **2** | **3** | **4** |
| GFP vs A6 | 2.31E-03 | 5.25E-04 | 1.31E-04 | 1.01E-03 | 2.85E-03 | 1.61E-03 | 1.16E-03 | 4.15E-05 | 8.66E-02 | 9.69E-02 |
| GFP vs A9 | 7.91E-01 | 1.38E-01 | 6.84E-01 | 8.25E-01 | 7.75E-01 | 6.52E-01 | 4.84E-02 | 7.63E-01 | 9.64E-01 | 7.65E-01 |
| A6 vs A9 | 6.39E-03 | 1.49E-03 | 6.82E-04 | 5.75E-03 | 2.28E-04 | 2.60E-03 | 3.58E-03 | 7.65E-04 | 1.12E-01 | 8.78E-03 |
| **ATP** | **0** | **5** | **Base** |  |  | **0** | **5** | **Base** |  |  |
| GFP vs A6 | 4.79E-03 | 7.19E-03 | 1.51E-02 |  |  | 7.70E-03 | 6.00E-03 | 1.68E-01 |  |  |
| GFP vs A9 | 7.03E-02 | 1.35E-01 | 3.74E-01 |  |  | 6.56E-01 | 5.75E-01 | 3.74E-01 |  |  |
| A6 vs A9 | 3.48E-03 | 4.68E-03 | 1.09E-02 |  |  | 2.22E-02 | 2.19E-03 | 4.05E-02 |  |  |
| **Citrate** | **0** | **1** | **2** | **3** | **4** | **0** | **1** | **2** | **3** | **4** |
| GFP vs A6 | 9.50E-04 | 1.50E-02 | 3.11E-04 | 3.72E-05 | 8.43E-04 | 1.17E-02 | 5.15E-01 | 1.95E-02 | 1.04E-04 | 8.64E-02 |
| GFP vs A9 | 4.28E-01 | 1.60E-01 | 1.18E-01 | 1.62E-01 | 3.20E-02 | 1.22E-01 | 4.84E-01 | 4.62E-02 | 2.74E-01 | 2.07E-02 |
| A6 vs A9 | 1.18E-03 | 8.61E-03 | 1.69E-04 | 1.57E-05 | 3.36E-04 | 1.81E-02 | 2.89E-01 | 8.46E-03 | 6.50E-03 | 1.50E-01 |
| **F6P** | **6** | **Scr** |  |  |  | **0** | **Scr** |  |  |  |
| GFP vs A6 | 1.49E-04 | 2.34E-04 |  |  |  | 1.12E-02 | 2.01E-03 |  |  |  |
| GFP vs A9 | 7.09E-03 | 4.09E-02 |  |  |  | 1.18E-01 | 1.13E-01 |  |  |  |
| A6 vs A9 | 3.58E-03 | 1.21E-02 |  |  |  | 4.07E-03 | 1.06E-01 |  |  |  |
| **Fumarate** | **0** | **1** | **2** | **3** | **4** | **0** | **1** | **2** | **3** |  |
| GFP vs A6 | 3.68E-03 | 3.70E-03 | 7.14E-04 | 1.31E-02 | 3.23E-03 | 1.06E-03 | 7.92E-02 | 2.19E-03 | 9.60E-02 |  |
| GFP vs A9 | 3.92E-01 | 9.09E-01 | 6.53E-01 | 5.30E-01 | 5.12E-01 | 8.79E-01 | 8.57E-01 | 9.06E-01 | 6.30E-01 |  |
| A6 vs A9 | 2.18E-02 | 1.84E-03 | 3.24E-03 | 9.72E-03 | 4.15E-04 | 2.36E-03 | 1.95E-02 | 1.61E-02 | 4.13E-02 |  |
| **G6P** | **6** | **Scr** |  |  |  | **0** | **Scr** |  |  |  |
| GFP vs A6 | 1.96E-05 | 5.02E-04 |  |  |  | 3.19E-02 | 8.56E-03 |  |  |  |
| GFP vs A9 | 2.02E-04 | 1.66E-02 |  |  |  | 3.78E-01 | 2.51E-01 |  |  |  |
| A6 vs A9 | 4.61E-05 | 1.03E-03 |  |  |  | 1.68E-03 | 1.25E-02 |  |  |  |
| **Glu** | **0** | **1** | **2** | **3** | **4** | **0** | **1** | **2** | **3** | **4** |
| GFP vs A6 | 1.42E-04 | 9.27E-04 | 8.30E-05 | 1.50E-01 | 3.20E-05 | 3.05E-02 | 3.97E-04 | 8.96E-03 | 2.24E-01 | 9.97E-04 |
| GFP vs A9 | 9.30E-01 |  | 7.40E-01 | 8.03E-02 | 4.35E-01 | 5.20E-01 |  | 8.58E-01 | 2.36E-01 | 1.58E-01 |
| A6 vs A9 | 6.13E-03 | 9.27E-04 | 1.75E-04 | 8.07E-01 | 8.60E-05 | 1.03E-02 | 3.97E-04 | 5.80E-06 | 5.75E-02 | 2.11E-04 |
| **GSH** | **0** | **2** | **3** | **4** |  | **0** | **2** | **3** | **4** |  |
| GFP vs A6 | 3.82E-02 | 3.82E-03 | 2.80E-03 | 3.87E-03 |  | 7.60E-02 | 8.29E-02 | 6.03E-02 | 5.26E-02 |  |
| GFP vs A9 | 7.88E-02 | 1.90E-02 | 1.64E-02 | 1.47E-02 |  | 7.34E-01 | 9.57E-01 | 5.90E-01 | 3.19E-01 |  |
| A6 vs A9 | 3.46E-02 | 5.27E-02 | 1.09E-01 | 1.25E-01 |  | 1.24E-02 | 2.95E-03 | 2.09E-01 | 9.50E-02 |  |
| **Inosine** | **0** | **5** | **Base** |  |  | **0** | **5** | **Base** |  |  |
| GFP vs A6 | 5.00E-02 | 1.71E-02 | 2.71E-02 |  |  | 3.31E-01 | 3.86E-01 | 1.75E-02 |  |  |
| GFP vs A9 | 7.24E-01 | 8.29E-02 | 1.78E-01 |  |  | 2.43E-01 | 2.18E-01 | 8.03E-02 |  |  |
| A6 vs A9 | 3.69E-02 | 3.00E-02 | 1.23E-01 |  |  | 2.78E-02 | 4.46E-03 | 2.58E-02 |  |  |
| **Lactate** | **0** | **3** | **Scr** |  |  | **0** | **3** | **Scr** |  |  |
| GFP vs A6 | 3.43E-03 | 1.96E-04 | 3.51E-05 |  |  | 4.32E-05 | 6.17E-05 | 1.54E-05 |  |  |
| GFP vs A9 | 6.36E-01 | 8.72E-01 | 9.88E-01 |  |  | 5.93E-01 | 5.78E-01 | 8.61E-01 |  |  |
| A6 vs A9 | 2.15E-02 | 6.16E-04 | 1.57E-04 |  |  | 6.57E-03 | 7.64E-03 | 1.33E-03 |  |  |
| **Malate** | **0** | **1** | **2** | **3** | **4** | **0** | **1** | **2** | **3** |  |
| GFP vs A6 | 1.20E-02 | 1.33E-03 | 7.85E-04 | 1.16E-03 | 3.80E-03 | 6.27E-03 | 3.24E-01 | 1.42E-04 | 1.02E-02 |  |
| GFP vs A9 | 8.73E-01 | 6.91E-01 | 9.69E-01 | 3.36E-01 | 1.66E-01 | 4.67E-01 | 4.96E-01 | 7.02E-01 | 1.13E-01 |  |
| A6 vs A9 | 1.53E-02 | 2.57E-03 | 1.66E-03 | 1.85E-03 | 9.84E-03 | 1.27E-04 | 6.76E-03 | 9.65E-04 | 4.98E-03 |  |
| **6PG** | **0** | **6** |  |  |  | **0** |  |  |  |  |
| GFP vs A6 | 1.22E-02 | 3.71E-01 |  |  |  | 1.65E-01 |  |  |  |  |
| GFP vs A9 | 2.56E-02 | 5.21E-03 |  |  |  | 2.33E-02 |  |  |  |  |
| A6 vs A9 | 7.08E-02 | 1.81E-01 |  |  |  | 7.36E-01 |  |  |  |  |
| **R5P** | **0** | **5** | **Scr** |  |  | **Scr** |  |  |  |  |
| GFP vs A6 | 3.15E-04 | 1.50E-04 | 2.46E-04 |  |  | 1.06E-02 |  |  |  |  |
| GFP vs A9 | 2.46E-01 | 1.53E-01 | 4.50E-01 |  |  | 7.75E-01 |  |  |  |  |
| A6 vs A9 | 4.47E-04 | 5.47E-04 | 2.84E-04 |  |  | 5.31E-03 |  |  |  |  |
| **Succinate** | **0** |  |  |  |  | **0** | **2** |  |  |  |
| GFP vs A6 | 6.23E-03 |  |  |  |  | 1.55E-02 | 6.25E-03 |  |  |  |
| GFP vs A9 | 9.77E-01 |  |  |  |  | 7.61E-01 | 7.70E-01 |  |  |  |
| A6 vs A9 | 3.47E-02 |  |  |  |  | 6.01E-03 | 2.69E-03 |  |  |  |
| **R1P** | **7** | **Scr** |  |  |  | **0** | **5** |  |  |  |
| GFP vs A6 | 1.17E-03 | 3.96E-02 |  |  |  | 1.09E-01 | 1.31E-02 |  |  |  |
| GFP vs A9 | 1.33E-02 | 3.40E-01 |  |  |  | 3.17E-01 | 4.91E-01 |  |  |  |
| A6 vs A9 | 4.00E-03 | 5.13E-02 |  |  |  | 2.16E-02 | 2.27E-02 |  |  |  |
|  | *p*-value (mmole/g protein) | |  |  |  |  |  |  |  |  |
| **Lactate_med_** | **3-^13^C** |  |  |  |  |  |  |  |  |  |
| GFP vs A6 | 1.93E-03 |  |  |  |  |  |  |  |  |  |
| GFP vs A9 | 7.64E-01 |  |  |  |  |  |  |  |  |  |
| A6 vs A9 | 8.40E-03 |  |  |  |  |  |  |  |  |  |
| **Glc_med_** | **1-^13^C** |  |  |  |  |  |  |  |  |  |
| GFP vs A6 | 8.77E-05 |  |  |  |  |  |  |  |  |  |
| GFP vs A9 | 9.28E-01 |  |  |  |  |  |  |  |  |  |
| A6 vs A9 | 8.97E-03 |  |  |  |  |  |  |  |  |  |

^1^ ^13^C isotopologues of metabolites listed are as in **Figs. 1**-**3**

^2^ *p*-values for metabolite amounts normalized to protein mass

^3^ *p*-values for fractional enrichment of unlabeled and ^13^C-labeled metabolites.

**Table S4. Student’s t test of control versus EPZ-6438 treatments in Fig. 4** ^1^

|  | *p*-value (fraction) | | | | | | |
| --- | --- | --- | --- | --- | --- | --- | --- |
| **ATP** | **0** | **DCxNx** | **N*Dx** | **C*NxDx** | **Total*** |  |  |
|  | 1.15E-02 | 7.44E-03 | 1.03E-02 | 1.06E-01 | 1.15E-02 |  |  |
| **F1,6BP** | **C*Dx** |  |  |  |  |  |  |
|  | 2.45E-02 |  |  |  |  |  |  |
| **F6P** | **0** | **DCx** | **C3Dx** | **C*Dx** | **Total*** |  |  |
|  | 1.09E-02 | 7.87E-03 | 7.64E-01 | 1.77E-02 | 1.09E-02 |  |  |
| **G6P** | **C3Dx** |  |  |  |  |  |  |
|  | 7.79E-02 |  |  |  |  |  |  |
| **Glu** | **N*CxDx** | **C5NxDx** |  |  |  |  |  |
|  | 4.05E-02 | 6.81E-02 |  |  |  |  |  |
| **GSH** | **0** | **DCxNx** | **N*CxDx** | **C5NxDx** | **C*NxDx** | **Total*** |  |
|  | 1.90E-02 | 2.31E-02 | 2.09E-02 | 3.04E-02 | 1.99E-02 | 1.90E-02 |  |
| **Inosine** | **0** | **D** | **N*Dx** | **C*NxDx** | **Total*** |  |  |
|  | 5.36E-03 | 1.61E-01 | 9.63E-03 | 7.52E-03 | 5.36E-03 |  |  |
| **Malate** | **0** | **C1Dx** | **C2Dx** | **C3Dx** | **C4Dx** | **C*Dx** | **Total*** |
|  | 2.50E-02 | 3.57E-02 | 8.10E-03 | 6.07E-02 | 3.67E-02 | 2.48E-02 | 2.50E-02 |
| **PEP** | **0** | **C3Dx** | **C*Dx** | **Total*** |  |  |  |
|  | 5.02E-02 | 1.33E-02 | 8.12E-02 | 5.02E-02 |  |  |  |
| **6PG** | **0** | **C*Dx** | **Total*** |  |  |  |  |
|  | 3.89E-02 | 6.15E-02 | 3.89E-02 |  |  |  |  |
| **UDP-GlcNAc** | **0** | **C*NxDx** | **Total*** |  |  |  |  |
|  | 2.44E-02 | 4.61E-03 | 2.44E-02 |  |  |  |  |
|  | *p*-value (µmole/g protein) | | | | | | |
| **ATP** | **0** | **DCxNx** | **N*Dx** | **C*NxDx** | **Total*** |  |  |
|  | 2.90E-02 | 5.69E-02 | 6.07E-02 | 4.90E-02 | 5.93E-02 |  |  |
| **Citrate** | **C1Dx** | **C2Dx** | **C3Dx** | **C4Dx** | **C*Dx** | **Total*** |  |
|  | 5.68E-02 | 3.81E-02 | 6.29E-02 | 3.15E-02 | 4.23E-02 | 3.73E-02 |  |
| **F1,6BP** | **0** | **C3Dx** | **Total*** |  |  |  |  |
|  | 5.54E-02 | 1.71E-02 | 9.54E-02 |  |  |  |  |

^1^ Isotopologues of metabolites listed are as in **Fig. 4**; *p*-values shown are for metabolites which displayed significant (p ≤ 0.05) or marginally significant (p ≤ 0.1) changes in response to EPZ-6438 treatment.

**Table S5. Student’s t test of control versus EPZ-6438 treatments in Fig. 5** ^1^

|  | *p*-value (fraction) | | | | |
| --- | --- | --- | --- | --- | --- |
| **Citrate** | **DCx** | **C2Dx** | **C3Dx** | **C5Dx** |  |
|  | 1.90E-03 | 7.35E-02 | 1.26E-02 | 4.22E-03 |  |
| **F1,6BP** | **C3Dx** | **C*Dx** |  |  |  |
|  | 2.20E-02 | 1.44E-02 |  |  |  |
| **F6P** | **C*Dx** |  |  |  |  |
|  | 1.64E-02 |  |  |  |  |
| **GAB** | **0** | **4-^13^C** |  |  |  |
|  | 3.28E-02 | 3.28E-02 |  |  |  |
| **GSH** | **C5NxDx** |  |  |  |  |
|  | 9.17E-02 |  |  |  |  |
| **IMP** | **DCxNx** | **C*NxDx** |  |  |  |
|  | 3.23E-03 | 4.17E-04 |  |  |  |
| **Inosine** | **0** | **DCxNx** | **N*CxDx** | **C*NxDx** | **Total*** |
|  | 6.19E-03 | 4.50E-03 | 3.89E-03 | 6.51E-03 | 6.19E-03 |
| **αKG** | **0** | **DCx** | **C5** | **C*Dx** | **Total*** |
|  | 1.88E-02 | 3.15E-02 | 1.23E-02 | 3.54E-02 | 1.88E-02 |
| **6PG** | **C3Dx** |  |  |  |  |
|  | 2.74E-02 |  |  |  |  |
| **S7P** | **0** | **DCx** | **C*Dx** | **Total*** |  |
|  | 4.15E-02 | 6.63E-02 | 2.08E-02 | 4.15E-02 |  |
|  | *p*-value (µmol/g protein) | | | | |
| **Citrate** | **DCx** | **C3Dx** |  |  |  |
|  | 1.62E-02 | 2.92E-02 |  |  |  |
| **F1,6BP** | **0** | **DCx** | **C3Dx** | **C*Dx** | **Total*** |
|  | 3.89E-02 | 2.96E-02 | 2.55E-02 | 3.18E-02 | 2.76E-02 |
| **GAB** | **4-^13^C** |  |  |  |  |
|  | 3.70E-02 |  |  |  |  |
| **GSH** | **DCxNx** | **N*CxDx** | **C5NxDx** | **C*NxDx** | **Total*** |
|  | 4.21E-03 | 4.13E-03 | 4.10E-03 | 4.20E-03 | 4.30E-03 |
| **Inosine** | **0** | **N*CxDx** |  |  |  |
|  | 8.45E-02 | 1.08E-01 |  |  |  |
| **αKG** | **0** | **C5** | **C*Dx** | **Total*** |  |
|  | 1.07E-01 | 7.95E-03 | 1.40E-02 | 1.41E-02 |  |
| **Malate** | **0** |  |  |  |  |
|  | 4.03E-02 |  |  |  |  |
| **PEP** | **0** | **DCx** | **C3Dx** | **C*Dx** | **Total*** |
|  | 6.03E-02 | 3.57E-02 | 5.13E-03 | 3.19E-02 | 3.35E-02 |
| **R1P** | **DCx** | **C*Dx** | **Total*** |  |  |
|  | 8.96E-04 | 4.60E-03 | 8.74E-04 |  |  |
| **S1,7BP ^2^** | **C*Dx** |  |  |  |  |
|  | 3.46E-02 |  |  |  |  |

^1^ Isotopologues or isotopomers of metabolites listed are as in **Fig. 5**; *p*-values shown are for metabolites which displayed significant (p ≤ 0.05) or marginally significant (p ≤ 0.1) changes in response to treatment.

^2^ t-test was done on protein-normalized MS intensity.
